# Traditional Food Environment and Factors Affecting Indigenous Food Consumption in Munda Tribal Community of Jharkhand, India

**DOI:** 10.3389/fnut.2020.600470

**Published:** 2021-02-01

**Authors:** Suparna Ghosh-Jerath, Ridhima Kapoor, Satabdi Barman, Geetanjali Singh, Archna Singh, Shauna Downs, Jessica Fanzo

**Affiliations:** ^1^Indian Institute of Public Health-Delhi, Public Health Foundation of India, Gurgaon, India; ^2^Department of Botany, Dr. Shyama Prasad Mukherjee University Ranchi, Jharkhand, India; ^3^Department of Biochemistry, All India Institute of Medical Sciences (AIIMS), New Delhi, India; ^4^Department of Urban-Global Public Health, Rutgers School of Public Health, Newark, NJ, United States; ^5^Berman Institute of Bioethics, Nitze School of Advanced International Studies (SAIS) and Bloomberg School of Public Health, Johns Hopkins University, Washington, DC, United States

**Keywords:** indigenous foods, traditional food environment, underutilized indigenous foods, nutritive value, micronutrients, factors affecting indigenous food consumption, Munda tribes

## Abstract

Indigenous food (IF) systems, derived from natural ecosystems are perceived to be sustainable and nutritionally adequate. Mundas, an indigenous tribal community in Jharkhand India, are surrounded by rich agroforestry resources, yet display high levels of malnutrition. Our study explored the food environment of Munda community, different IFs they accessed, levels of utilization of IFs in routine diets, their nutritional attributes and factors influencing IF consumption. A cross-sectional mixed-methods study was conducted in nine villages of Murhu and Torpa blocks in Khunti district, Jharkhand. Using focus group discussions and key informant interviews, we did free-listing of IFs known to the community. This was followed by enumerating preferred and little used/historically consumed IFs, along with reasons. Qualitative enquiries were recorded and transcribed verbatim; data were coded and analyzed using thematic framework approach. The listed IFs were identified through common names and photographs, and verified by ethnobotanist in the team. The nutritive values of identified IFs were searched in literature or nutritional analysis of specific plant based foods were undertaken in an accredited laboratory. The community demonstrated traditional ecological knowledge of several IFs (*n* = 194), which are accessed from wild, cultivated and built food environments. Taxonomic classification was available for 80% (*n* = 156) IFs, out of which 60 foods had nutritive values in secondary literature and 42 foods were analyzed in laboratory. Many IFs were rich in micronutrients like calcium, iron, folate, vitamin A and C. Among the listed IFs, only 45% were commonly consumed, while rest were little used/historically consumed. Factors like desirable taste, satiety, perceived nutrition benefits, adaptability to climate variability, traditional practice of food preservation and their cultural importance promoted IF consumption. However, local climatic impacts on agroforestry systems, easy access to foods bought from markets or distributed under government food security schemes, and promotion of hybrid seeds by local agricultural organizations, emerged as potential barriers. Thus, reinforcement of traditional ecological knowledge and informal food literacy, along with promotion of climate resilient attributes of IFs, can contribute to sustainable food systems in Munda community.

## Introduction

Food systems comprise of elements and activities that relate to the way in which the food is produced, processed, distributed, prepared, and consumed (1). The interface where people interact with the wider food system to acquire and consume foods is defined as the food environment (2, 3). Depending on geographical location, people interface with wild, cultivated and built (i.e., market) food environments (4). The attributes of foods within these environments influence peoples' food choices and has the potential to affect their nutritional status (5). Sustainable Development Goal (SDG) 2 promises to end hunger, achieve food and nutrition security, and promote sustainable agriculture among all populations, especially nutritionally vulnerable people (6). However, achieving this goal is riddled with uncertainty because of the way in which the world currently produces and consumes foods. Globally, the diets we consume and the food systems that produce them, are neither healthy nor sustainable, which has implications for achieving SDG 2. Despite being reasonably safe and consistent in food supplies (quantity), current global food systems are struggling to meet the nutritional needs of the growing population and have placed significant strain on land, water, soil, air, and other natural resources (7, 8). In this context, the concept of sustainable food systems and healthy diets are receiving renewed attention (9).

Sustainable food systems are those that aim at achieving food and nutrition security while limiting negative environmental impacts and improving socio-economic welfare of all, including poor and marginalized populations (7). These sustainable food systems are derived from sustainable cultures and ecosystems, and are accessible, affordable, safe and healthy, while simultaneously promoting environmental stability (8, 10). Indigenous foods, accessed as part of traditional food systems and consumed by indigenous people throughout the world, are also derived from natural ecosystems, and are hence, perceived to be sustainable (11). These food systems are reservoirs of unique traditional ecological knowledge, incorporated in both cultivated and wild foods derived from plants, animals, and fungi species that are available from local natural resources. Moreover, indigenous food systems are better adapted to local conditions, more resistant to drought, altitude, flooding, or other extreme conditions, are low resource intense, have low carbon footprints and use environmentally sensitive technologies (12). Several indigenous foods accessed as part of these food systems are known to be nutrient rich and may have potential in alleviating hunger and malnutrition (10, 13). The indigenous food systems are grounded in historical legacy and spirituality that acknowledge the inextricable link of people with their sustainably managed resources, and thus can be utilized to provide sustainable diets and can play a crucial role in achieving SDG 2 (7, 8).

Indigenous people, despite their vast knowledge of the world's territories and guardianship of 80% of global species diversity, are nutritionally vulnerable and experience significant disparities in health outcomes, grounded in poverty and marginalization (14). This contributes to their inability to realize the potential of their traditional food systems in providing sustainable solutions to the existing nutrition insecurity within the population (15). Factors like declining traditional knowledge, opportunity cost in access, displacement of traditional crop species by a few major crops and shifting diets and food cultures have substantially influenced their food systems (16) and led to underutilization of many diverse indigenous food resources (17, 18). Nonetheless, several factors have been documented in the scientific literature to promote these food systems such as local accessibility, cost effectiveness, existence of traditional knowledge, sustainable utilization of the natural environment from farming or wild harvesting and socio-ecological resilience (19–22).

The tribal communities in India are a good example of indigenous populations, having their own rich cultural and social traditions and unique indigenous food systems (23). Jharkhand, a central eastern Indian state known for its rich biodiverse agroforestry (24, 25), is home to several indigenous tribal communities that constitute 26.2% of the state's population (26). Mundas, the third most populous tribal community of Jharkhand, are the inhabitants of Chota Nagpur region in the state (27). This community lives in geographical locations that are surrounded by natural resources and have largely retained their traditional ecological knowledge (TEK) of gathering and preparing foods from the natural sources in traditional ways (28). They are subsistence farmers (29) and are also known to use local wild plants, fruits and tubers which could help to augment their food and nutrition security (28, 30). Despite access to a rich agroforestry, factors like geographical isolation, poverty, lack of formal education and poor access to health services contribute to poor nutrition outcomes in Munda community (31). Although limited literature is available on the nutritional status of Mundas, a few studies have documented high prevalence of malnutrition among women and children (32, 33). In a cross-sectional study conducted among Munda children, more than half the children (56%) were found to be underweight, with 29% children as severely underweight (32). The nutrient intakes among Munda women were found to be below recommended levels of Indian women, especially for nutrients like protein, calcium, vitamin A and C, thus indicating consumption of poor quality diets (33). Hence, the aims of this study were to explore the traditional food environment of the Munda tribal community of Jharkhand, examine their TEK and nutritive value of indigenous foods and the factors that influence their consumption.

## Materials and Methods

### Study Locale and Population

**Figure 1:**
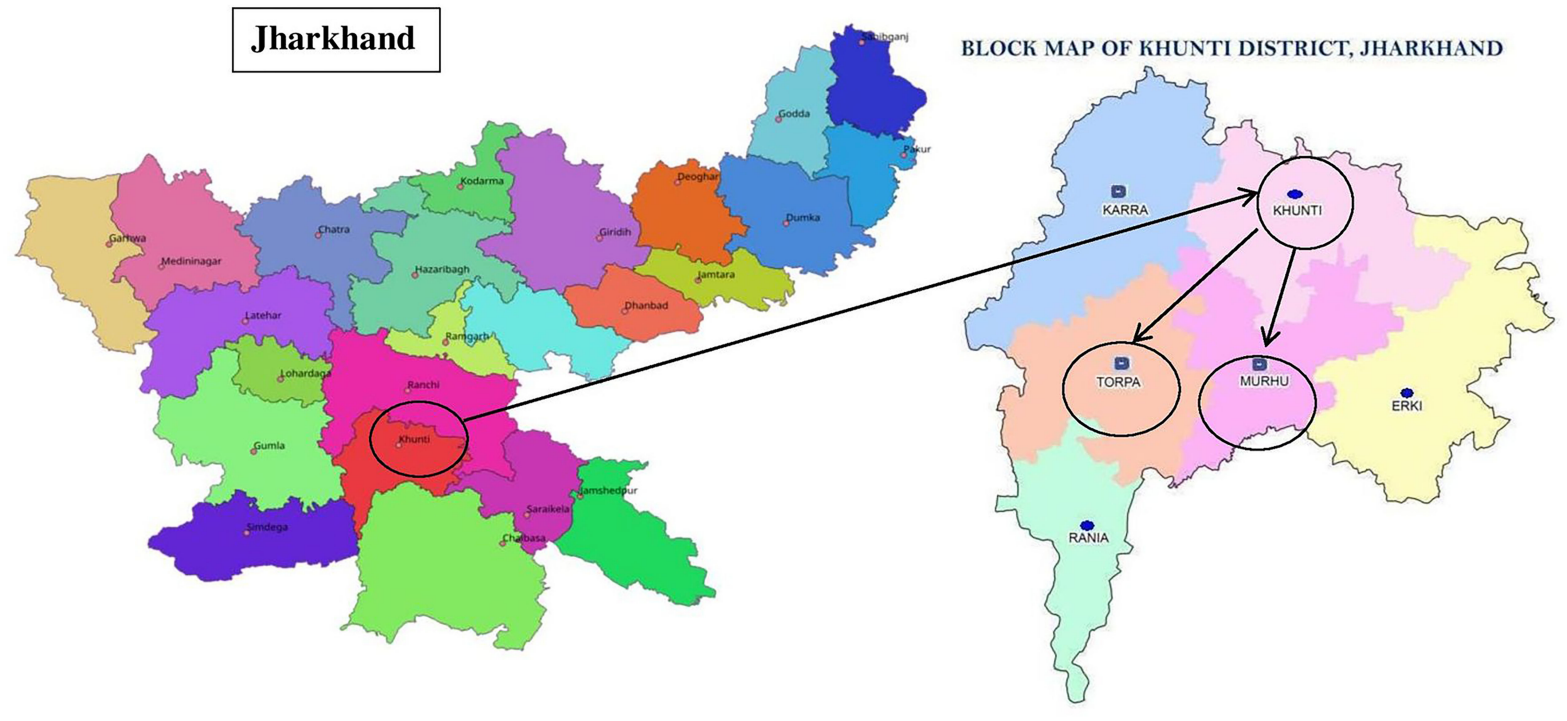
Selection of Murhu and Topra blocks from Khunti district of Jharkhand, India.

This study was conducted in Khunti district of Jharkhand, India. The total area of Khunti district is about 2,535 sq. km (34) and 40% of it is covered by forests and uneven landscapes (28). The total population in the district is 531,885, out of which 61% is comprised of the Munda tribal community (34). Two geographically distinct blocks of Khunti, namely, Murhu and Torpa were purposively selected for the study (Figure 1). Both these blocks comprise of hilly terrain with forest cover and plain lands, and have a predominantly higher population of Munda tribe, in comparison to other blocks. Using probability proportional to size sampling, a total of eleven villages from Murhu and Torpa blocks were selected for the study, details of which are reported elsewhere (35). Out of these villages, qualitative enquiries were conducted in nine villages until theoretical saturation was achieved (i.e. no additional information relevant to the research question was generated) (36).

### Study Design

A cross-sectional mixed-method study was conducted using both qualitative enquiries and quantitative estimates, to explore the traditional food environment of the Munda tribal community, including the types of food environments (wild, cultivated, and built) that they interface with, the types of indigenous foods (IFs) that are accessed, their nutritional attributes as well as the factors influencing the consumption of these foods. This work was part of a larger study documenting the role of IFs consumed by tribal groups of Jharkhand and assessing their contribution to food security and dietary diversity among women and children (35). The data collection was conducted between June 2019 and January 2020; multiple visits were conducted to capture the diversity of foods that were consumed during different seasons.

### Study Procedures

**Figure 2:**
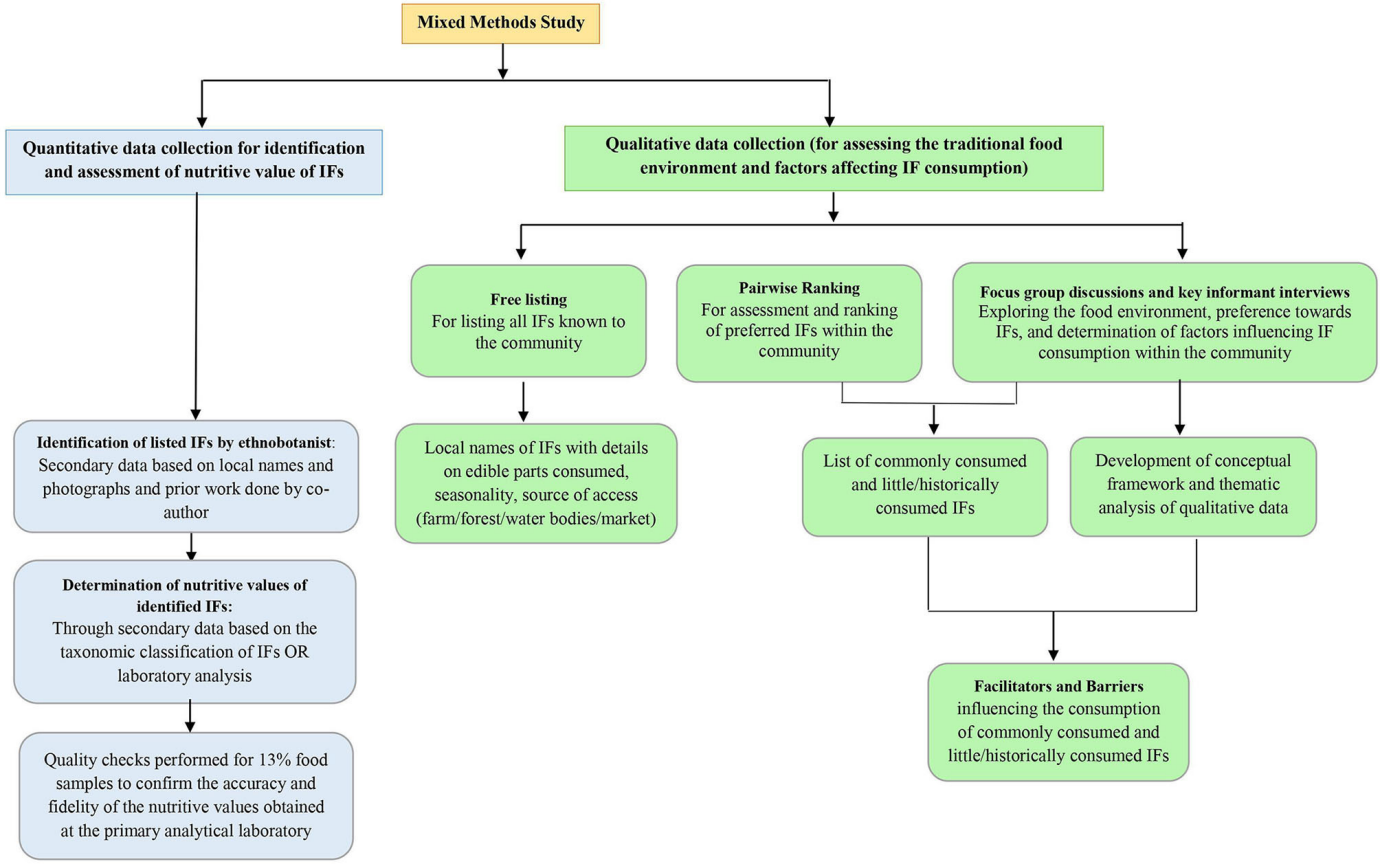
Detailed flow chart on the methodological approach used in the study.

The study included both qualitative and quantitative methods of data collection. The mixed methods approach facilitated the triangulation of both qualitative and quantitative data to increase the data integrity and provide rich contextual information (37). A detailed flow chart on the methodological approach used in the study is provided in Figure 2.

Qualitative enquires were conducted using focus group discussions (FGDs) and key informant interviews. A total of nine FGDs and six interviews were conducted in nine study villages using FGD and interview guides that were pretested in three villages (other than the study villages). The FGD guides were used to do free-listing of all IFs known to the community, and gain information on their traditional farming methods, food collection from natural sources like forests and water bodies, access to market, usual eating patterns and cultural use of IFs. The impact of climate change on local food systems was also assessed by adapting questions from the tool developed by Bioversity International and the Institute of Development Studies (38). After developing a free list of all the IFs known to the community, participants were further probed in order to develop the list of popular and preferred IFs vs. those that were little used or historically consumed. A pairwise ranking method (39) was used to elicit further information on the preference toward specific IFs in a food group category among the list of preferred foods in a specific season. The perceptions on reasons why specific IFs were preferred or infrequently used (e.g., availability, access/production, and/or taste) and other factors that influenced IF consumption were also explored. Based on certain queries and information gaps from FGD, additionally six key-informant interviews were conducted to substantiate and cross-compare the findings.

The listed IFs were identified through their common names as well as photographs and verified by an ethnobotanist in the team, who has extensively worked on taxonomic classification of Munda tribal foods (28, 30, 40–47). The nutritive value of these identified IFs were searched in the Indian food composition database and other secondary literature (23, 40, 42–45) and were collated. IFs with no secondary data on nutritional values, were collected from field sites and sent for analysis to a food testing laboratory accredited with National Accreditation Board for Testing and Calibration Laboratories (NABL). The food sample collection was carried out following the standard protocol developed as part of the larger study (35) and nutrient analysis was conducted according to standard reference procedures in consultation with the NABL accredited laboratory. The parameters analyzed included energy, protein, carbohydrate, fat, dietary fiber, vitamin A (as beta-carotene), vitamin C, vitamin B1, B2, iron, calcium, zinc, folate, and phosphorous. The analyte values were reported per 100 g of edible weight. The details of the methods used for specific nutrients and the limit of quantification are provided in Supplementary Table 1.

The entire data collection was conducted and supervised by the core research team. In addition to the core research team, the study team also included well-trained local field workers fluent in the native *Mundari* dialect who assisted the team in facilitating the qualitative enquiries and collection of food samples for identification and nutritional analysis purposes. Two local field workers were given a two-day training prior to the FGDs and interviews wherein they were briefed about the study objectives and their role as translators during the qualitative enquiries. Three workers were also trained regarding the steps of collection, packaging and transportation of food samples from the field to the ethnobotanist's laboratory and food analysis laboratory.

### Study Participants

The FGDs were attended by village members from different age groups such as young adults as well as elderly men and women, along with community health and nutrition workers (*Anganwadi* worker and Accredited Social Health Activist, ASHAs) and community leaders. Village wise details on age and gender characteristics of study respondents are listed in Table 1. The community health workers and village heads were contacted prior to the FGDs and were asked to inform the village members about the scheduled discussion. Sample selection was based on snowball sampling method (48); wherein study participants were asked to identify other potential community members who had traditional knowledge on indigenous foods, their accessibility, use and other related information. Key informant interviews were conducted with community leaders and/or health and nutrition workers in the villages in order to substantiate and fill some critical information gaps on IF preferences and reasons for consumption /non-consumption gathered during the FGDs.

**Table 1:** Characteristics of study participants in villages of Murhu and Torpa blocks, Khunti district, Jharkhand.

**Block**	**Study Village**	**Group size**	**Gender and age group of respondents**
Block 1: Murhu	Charid	10	10 adults (women)
	Gangina	6	1 elderly (woman), 5 adults (4 men and 1 woman)
	Burju	7	1 elderly man, 6 adult men
	Kurki	11	1 elderly woman, 10 adult women
Block 2: Torpa	Urikel^*^	13	13 adults (7 men and 6 women)
		9	2 elderly men, 7 adult men
	Tati	17	15 adults (8 men and 7 women), 2 elderly women
	Nichitpur	24	5 elderly (2 men and 3 women), 19 adults (10 men and 9 women)
	Jibilong	14	1 elderly man, 13 adults (6 men and 7 women)

* *Two separate FGDs were conducted in two different Tolas (hamlets) of the village*.

### Data Analysis

All the FGDs and interviews were recorded and transcribed from *Mundari* to Hindi and then translated to English. The transcripts were used to generate a list of all the IFs known to the community. Further, based on the inputs from the participants, the listed foods were categorized into commonly consumed and little consumed/historically consumed IFs. The information from pairwise ranking was used to assess preference toward specific IFs in the preferred food list category. This was done by creating comparisons and scoring between IFs under each food group. The nutritive value of IFs were documented and utilized to prepare a list of IFs that are “good” and “rich” nutrient sources. The foods with at least one nutrient level between 10 and 19% and higher than 20% of recommended dietary allowances (RDA) for Indian adults per serve, were considered as “good” and “rich” sources of nutrients, respectively (49–51). This was done for both commonly consumed and little used/historically consumed IFs. Atlas.ti version 8 was used for coding the content of the transcripts, which was analyzed further using thematic analysis (52). The data were coded using deductive approach; similar codes were identified and merged to produce main themes and sub-themes related to factors affecting IF consumption. A conceptual framework was then developed based on the analysis of qualitative enquiries and seven main themes were generated which highlighted factors that directly or indirectly influenced consumption of IFs within the community.

### Ethics Approval

The study was conducted according to guidelines laid down in Declaration of Helsinki (53) and all procedures involving humans in this study were approved by the Institutional Ethics Committee at Indian Institute of Public Health-Delhi, Public Health Foundation of India, and All India Institute of Medical Sciences, New Delhi. Administrative approvals from authorities at district level and cluster level consent from the village leaders were obtained before conducting FGDs. Written informed consent was obtained from literate respondents and third-party witnessed verbal consents were sought from illiterate respondents. All respondents were informed that the FGDs and interviews were being recorded.

## Results

### Traditional Food Environment of Munda Tribal Community

The community reported a predominantly rice based habitual diet with most of the households consuming two to three main meals a day. The usual meals consist of rice along with sautéed or curried green leafy vegetables (GLVs) or roots and tubers or sometimes pulses and flesh foods (meat, poultry, egg, or fish). Both indigenous as well as non-indigenous varieties of foods are consumed, which comprise of different varieties of rice, pulses, fruits, GLVs, roots and tubers, vegetables, and flesh foods (detailed information on individual food items are added in later sections). Milk and milk products are rarely consumed by the community. The community practices smallholder subsistence agriculture and accesses foods from cultivated (agricultural lands, backyard gardens and raising livestocks), wild (surrounding forests, pastures, roadsides, wastelands and local water bodies), and built food environments (local informal markets and food entitlements under government's food security programmes). The agroforestry and livestock produce is mainly utilized for household consumption, while the surplus is sold in local markets for income generation. We have described the food environments as well as the specific IFs accessed within them in additional detail below.

#### Cultivated Food Environment

The Munda community mainly practices settled agriculture at three levels of farm lands, namely-*Loyong* (low level lands with highest water requirement for crops), *Badi* (middle level lands with relatively low water requirement for crops), and *Godha* (dry stony plain lands with least water requirement). The usual size of farmlands varies between 1.5 to 3 Bigha (traditional unit of land measurement, equivalent to 0.25 Hectares). The crops that are commonly cultivated include both indigenous as well as hybrid varieties of rice, millets, and pulses (details given in later sections). However, the proportion of land use reported for cultivation of hybrid varieties is substantially larger than indigenous varieties. In addition to their farm lands, they also grow vegetables, roots and tubers in their backyard kitchen gardens, locally known as *Bakdi*. The community also raises livestock such as goat, pig, and poultry to produce commodities such as meat and eggs.

#### Wild Food Environment

The community accesses the wild food environment, including forests and water bodies and natural vegetation like fields and pastures within the village. Most of the study villages reported accessing local forests for gathering forest foods as well as firewood. Different varieties of indigenous leafy vegetables, fruits, roots and tubers, and mushrooms (details discussed in later sections) are collected for the purpose of both household consumption and sale in local markets, while collected firewood is used as a household cooking fuel. The practice of hunting was also reported, wherein all men in the village gather once in a year and collectively hunt the wild animals for consumption. Local rivers, lakes and ponds are accessed, especially during monsoons, for collection of indigenous fishes, crabs, and snails. In addition to these, weeds grown in the field, pastures and wastelands are also collected for consumption.

#### Built Food Environment

The community frequently accesses weekly local informal markets or “*Hatiya*,” which are situated within a range of 5–10 kilometers from the villages. These local markets are mainly accessed for procurement of cooking oil, spices, packaged foods, and freshly prepared sweets and savories. Apart from this, the local markets also provide the community with indigenous as well as non-indigenous varieties of pulses, fruits, vegetables, and roots and tubers, meat and fish (details discussed in later sections). Specific varieties of indigenous foods accessed from local markets include pulses [Munmuna (“*Baturi dal”: Vicia hirsuta)*, Horse gram (“*Kulthi”*: *Macrotyloma uniflorum)*], vegetables [Cowpea, white (“*Bodi”*: *Vigna unguiculata*), Field beans (“*Simbi”: Lablab purpureus)*] and roots and tubers [Pechki (“*Toti”: Colocasia esculenta), “Jat sanga”* (*Dioscorea alata)*].

In addition to the weekly markets, households in all study villages receive subsidized food commodities (rice, sugar, salt etc.) under the Public Distribution System (PDS) – a government food security scheme (54). The children under 6 years, receive supplementary nutrition, mostly in the form of hot cooked meals and take home ration from *Anganwadi* centers (maternal and child health and nutrition center) under Integrated Child Development Service (ICDS) (55), while school children receive cooked meals under the Mid-Day Meal (MDM) program (56).

### Indigenous Foods of Munda Tribal Community

The community was asked to list out all IFs known. This was followed by identifying IFs that are routinely consumed and those that are little used or historically consumed.

#### Types of IFs Based on Free Listing

The FGDs revealed a rich TEK within the community which resulted in a diverse list of 194 IFs, comprising of 34 cereals (17.5%), 7 pulses (3.6%), 57 green leafy vegetables (GLVs) (29.2%), 11 other vegetables (5.7%), 9 roots and tubers (4.7%), 15 fruits (7.8%), 23 mushrooms (12%), 37 flesh foods (19%), and honey. Supplementary Table 2 provides a list of all the IFs mentioned by the study participants with details of the parts consumed (in case of plants), their primary sources from where they are harvested, gathered, or collected and the season in which they are available.

#### Classification of IFs Based on Preference

**Figure 3:**
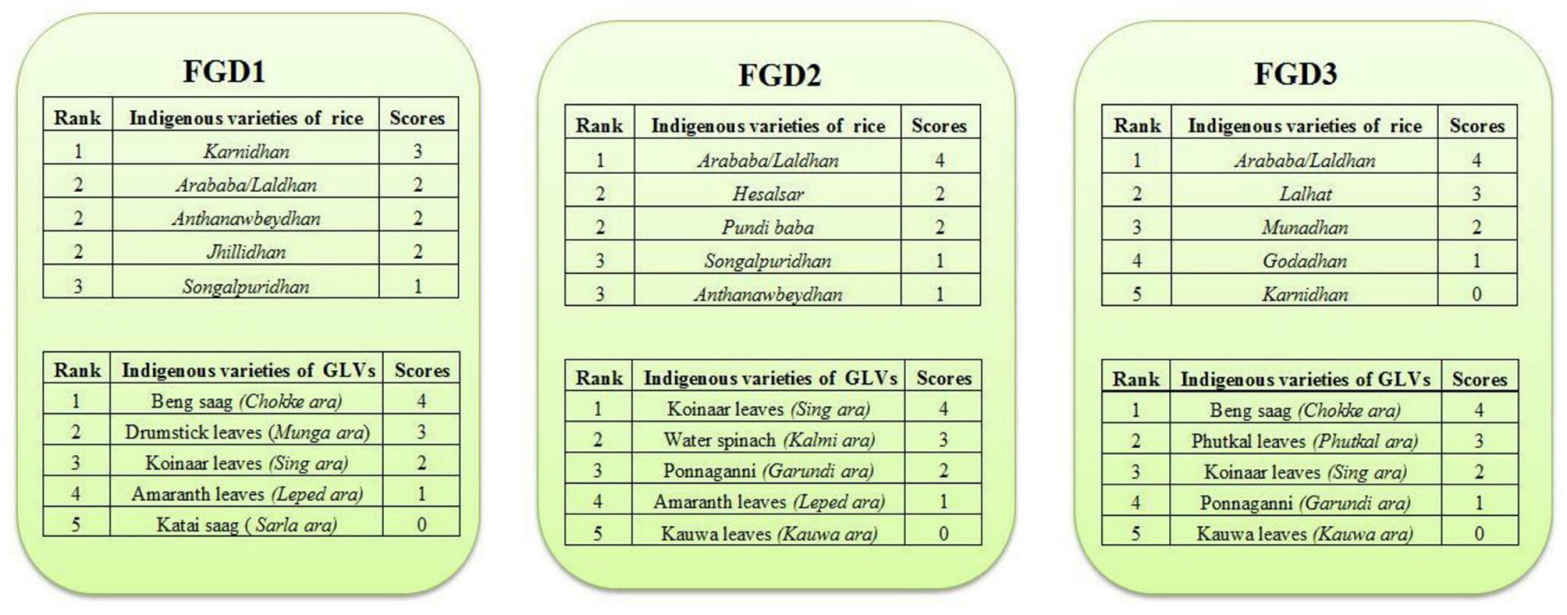
Pairwise ranking and scores to indigenous foods under different food groups. Note: GLVs, Green Leafy Vegetables.

Out of 194 IFs listed, 87 (45%) were identified as commonly consumed and 107 IFs (55%) as little used or historically consumed. Pairwise ranking method, based on criterion such as taste, availability, ease of production or collection by the community, further revealed comparative food group wise preference toward specific IFs from the preferred food list category for a specific season. Figure 3 provides some examples of scoring and ranking of indigenous rice and GLVs. To elaborate, the FGD participants were asked to identify 4 to 5 most preferred indigenous rice varieties which were then tabulated as a matrix on a flip chart. Participants were asked to compare the first rice variety in the row with various others in the column one by one. This step was repeated for the subsequent rice varieties listed in the columns. A score was provided based on the number of times a specific rice variety was selected, and then the varieties were ranked. The rice variety with the highest scores (“*Laldhan”*: *Oryza sativa*) was ranked first and the successive scores in descending order provided information on comparative preferences toward other varieties. This helped in identifying the popular IFs among the commonly consumed IFs under different food groups. Detailed list of the preferred and little used/ historically consumed IFs are provided in Supplementary Table 2.

Rice is the primary cereal consumed, though other cereals like Maize (“*Jondra”: Zea mays)*, Finger millet (“*Kodde”*: *Eleusine coracana*), Pearl millet (“*Gaangi”: Pennisetum glaucum)*, Sorghum (“*Jowar”*: *Sorghum bicolor)*, and Little millet (“*Gondli”*: *Panicum Miliare)* were also mentioned during the FGDs. About 29 varieties of indigenous rice were mentioned, but only 14 were reported to be routinely consumed. Millets are presently little used or historically consumed. Out of 57 GLVs reported, only about half are routinely consumed. There were several mushrooms (*n* = 23) listed, however, only 12 varieties are routinely consumed. Similarly, only about 50% of roots and tubers (5 out 9) and fruits (7 out of 15) are routinely consumed at present. Only a third of the reported 37 animal foods are included in the routine diets.

**Figure 4:**
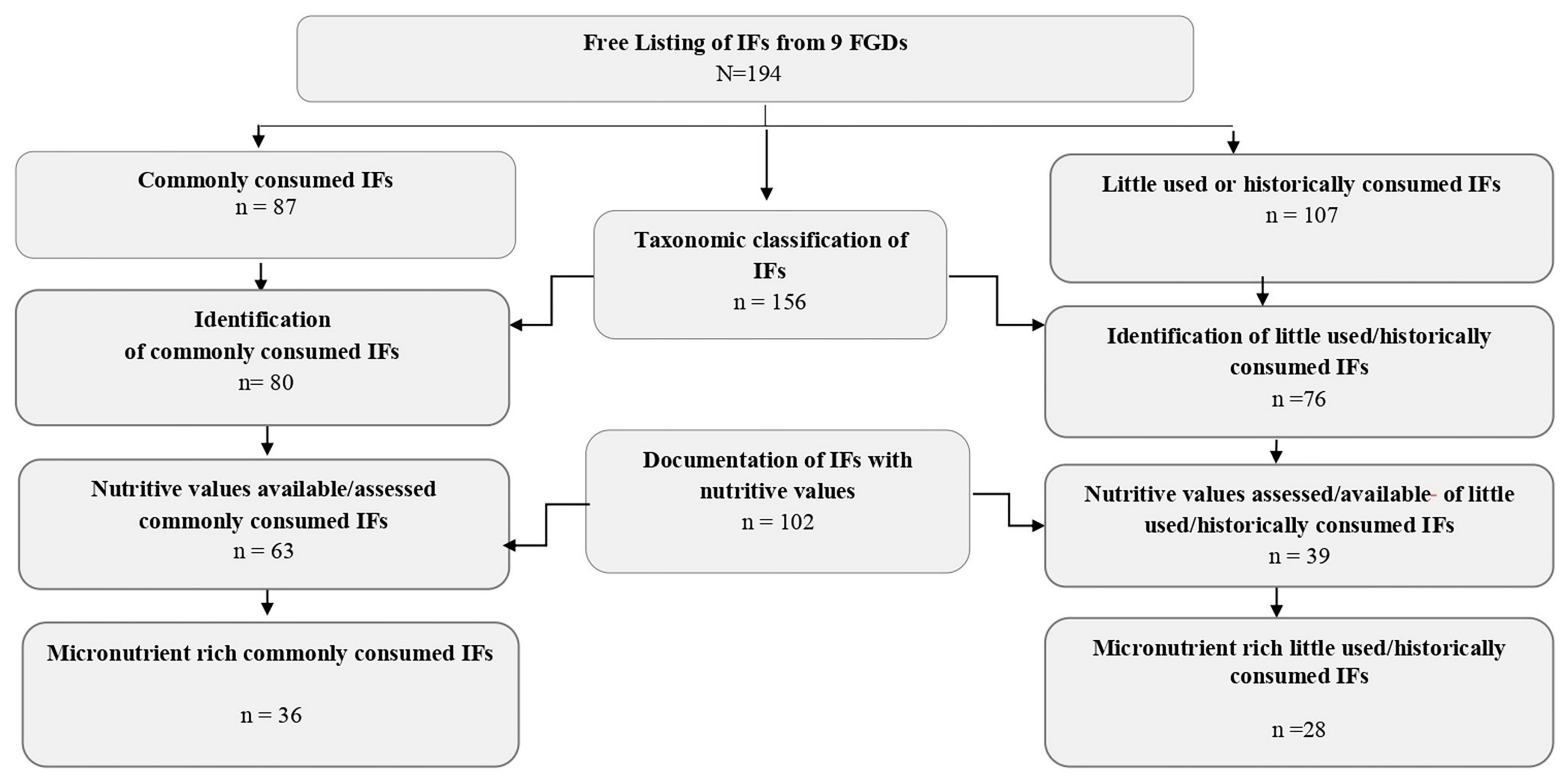
The systematic approach adopted to classify IFs available in the community.

A systematic documentation of total number of identified IFs (preferred and little used/historically consumed), the status of their taxonomic classification and assessment of their nutritive values are provided in Figure 4.

#### Taxonomic Classification

A total of 156 IFs (80%) were identified with taxonomic classification, that comprised of 80 (51%) commonly consumed foods and 76 (49%) little used/historically consumed foods. Details on taxonomic classification are provided in Supplementary Table 2.

#### Nutritive Values of IFs

Based on the taxonomic classification of IFs, the nutritive values of 102 IFs were documented (63 commonly consumed IFs and 39 little used/historically consumed). Out of these, nutritive values were available for 60 IFs from secondary sources while the rest were assessed in laboratory as the part of the study (*n* = 42), Supplementary Table 3.

**Figure 5:**
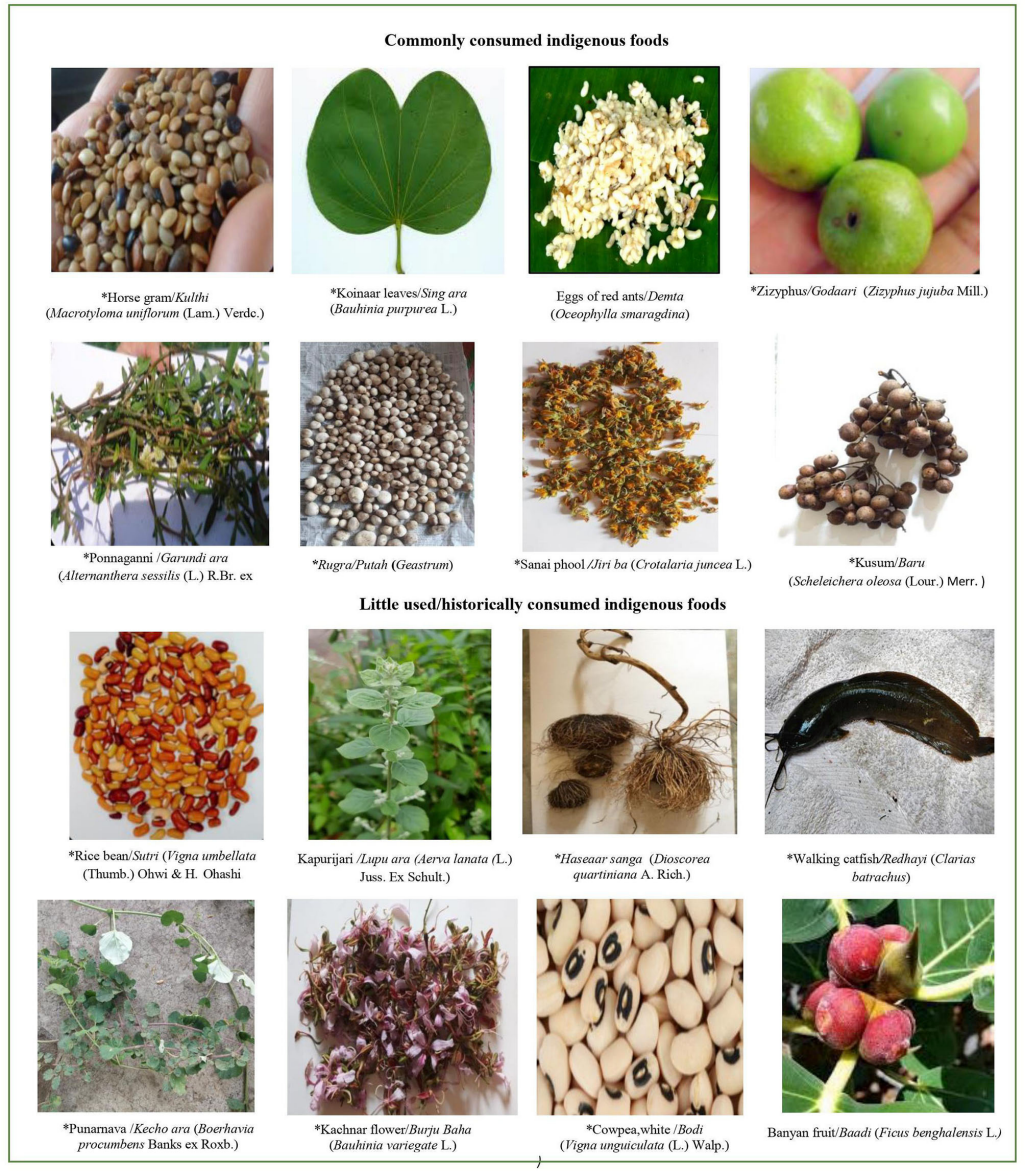
Some micronutrient rich indigenous foods of Munda tribal community. Picture courtesy: ^*^Indigenous Foods project, DBT/Wellcome Trust India Alliance.

Many IFs were found to be good sources of micronutrients (Table 2). Among indigenous cereal varieties, Finger millet (*Kodde*), a little consumed IF, was found to be a good source of calcium (364 mg/100 g). Indigenous pulses were not only found to have good levels of protein (range 21.3–28.2 g/100 g), but were also rich in several micronutrients. For instance, Horse gram (*Kulthi*), a commonly consumed pulse, was found to be a good source of calcium (269 mg/100 g) as well as folate (163 μg/100 g). Little consumed pulses were also found to be good source of micronutrients; for example, Rice bean (“*Sutri”*: *Vigna umbellata*) had high levels of calcium (302 mg/100 g), Cow pea, white (*Bodi*) had high levels of folate (249 μg/100 g) and Munmuna (*Baturi dal*) was rich in iron (17.1 mg/100 g). Most of the GLVs were found to be rich sources of vitamin A (range: 1015 to 21760 μg/100 g), with the highest content observed in Kapurijari (“*Lupu ara”: Aerva lanata*), an under-utilized GLV. The commonly eaten Ponnaganni leaves (“*Garundi ara”: Alternanthera sessilis*) were found to have remarkably high levels of vitamin C (103 mg/100g). Maximum calcium content among GLVs (877.9 mg/100 g) was seen in Punarnava (“*Kecho ara”: Boerhavia procumbens*), while highest iron content (81.1 mg/100 g) was seen in Kantha leaves (“*Kantha ara”: Dentella repens*), both little consumed within the community. Some GLVs were found to have ideal calcium to phosphorous ratio of around 2:1, which is important for bone health. Flowers of Kachnar (“*Burju Baha”: Bauhinia variegate*) (little consumed) and Sanai (“*Jiri ba”*: *Crotalaria juncea)* (frequently consumed), which are eaten as vegetables, were found to be rich in calcium (404.9 mg/100 g) and vitamin A (1113 μg/100 g), respectively. Among roots and tubers, under-utilized varieties of Tapioca (“*Adel sanga”: Manihot esculents*) was found to be rich in vitamin C (17.6 mg/100 g), while “*Haseaar sanga” (Dioscorea quartiniana)* was found to be iron-rich (55.9 mg/100 g). Little consumed fruits such as Marking nut (“*Soso”: Semecarpus anacardium*) and Banyan fruit (“*Baadi”: Ficus benghalensis*) were found to have high calcium content (295–364 mg/100 g). Marking nut was also found to be exceptionally rich in protein (26.4 g/100 g). Fruit of Kusum (“*Baru”*: *Schleichera* oleosa) which is frequently consumed in summers, was found to be rich in vitamin A (6238 μg/100 g), calcium (134.8 mg/100 g), and iron (44.2 mg/100 g). Highest value of vitamin C (60.9 mg/100 g) was observed in Zizphus (“*Godaari”: Zizyphus jujuba*), which is frequently consumed during winters. Commonly consumed mushrooms such as, “*Rugra/Putuh” (Geastrum)*, was found to be a rich source of calcium (193.4 mg/100 g), while *Gitilud* (classification NA) was found to be rich in iron (10.8 mg/100 g). Most of the commonly consumed flesh foods were found to have high content of calcium (range 104 to 870 mg/100 g). Among little consumed flesh foods, Walking catfish (“*Redhayi”: Clarias batrachus*) was found to be a good source of calcium (210 mg/100 g). Pictures of some micronutrient rich indigenous foods are provided in Figure 5.

**Table 2:** ^**^Indigenous foods with high micronutrient content.

**S. No**.	**Food item (Mundari Name)**	**Common name (English/ Hindi)**	**Scientific name^β^**	**Energy (Kcal/ 100 g)**	**Protein (g/100 g)**	**Carbohydrate (g/100 g)**	**Fat (g/100g)**	**Dietary fiber (g/100g)**	**β- Carotene/Retinol (μg/100g)^**a**^**	**Vit C (mg/100g)**	**Vit B1 (mg/100g)**	**Vit B2 (mg/100g)**	**Iron (mg/100g)**	**Zinc (mg/100g)**	**Calcium (mg/100g)**	**Total folate (μg/100g)**	**Phosphorus (mg/100g)**
**Commonly consumed Indigenous foods**																	
1	*Pundigoda*^¥^	Rice	*Oryza sativa* L.	351	4.28	80.7	1.26	5.19	ND	ND	0.44	0.12	0.4	0.1	20.6	3	37.6
2	Kulthi^*^	Horse Gram, whole	*Macrotyloma uniflorum* (Lam.) Verdc.^€^	321	22	57.2	0.5	NA	59	NA	0.32	0.24	8.8	2.7	269	163	298
3	Garundi/ Gundri ara^*^	Ponnaganni	*Alternanthera sessilis* (L.) R.Br. ex DC.	51	5.3	5.17	0.71	6.74	5288	103	0.02	0.1	3.9	1	388	48	53.2
4	*Jiri ba*^¥^	Sanai phool	*Crotalaria juncea* L.	120	2.9	27.04	ND	7.43	1113	1.77	3.09	ND	7.6	0.15	320.2	ND	537.2
5	Ber^*^	Zizyphus	*Zizyphus jujuba* Mill.	49	1.3	9.4	0.35	3.73	2	60.9	0.01	0.02	0.4	0.1	46.6	6	32.3
6	*Dahu*^¥^	Barhar	*Artocarpus lakoocha* Wall. Ex Roxb.	121	2.88	27.33	ND	7.2	1843	8.9	0.32	1.32	1.8	0.1	54.7	ND	ND
7	*Kusum/ Baru*^¥^	Kusum fruit	*Schleichera* oleosa (Lour.) Merr.	144	6.31	29.72	ND	14.89	6238	3.1	ND	ND	44.2	0.8	135	ND	51.3
8	*Rugra/ Putuh*^¥^	Mushroom	*Geastrum*	138	4.86	29.53	0.06	7.37	ND	ND	0.58	0.37	6.8	3.1	193	0.2	30.2
9	*Gitilud*^¥^	Mushroom	-	67	3.52	13.17	ND	6.89	9	ND	0.19	0.17	10.8	0.6	10	3	24.3
10	*Loa suti*^$^	Snail	*Pila globosa*	97	10.5	12.4	0.6	NA	NA	NA	NA	NA	NA	NA	870	NA	116
11	*Setua/ Keyosuti*^$^	Mussel	*Margaritifera margaritifera*	81	14.5	2.1	1.6	NA	NA	NA	NA	NA	NA	NA	592	NA	NA
12	*Demta/Hau anda*^$^	Eggs of red ants	*Oceophylla smaragdina*	131	13.4	9.1	4.6	NA	NA	NA	NA	NA	NA	NA	104	NA	107
**Little used/historically consumed Indigenous foods**																	
1	Kodde^*^	Finger millet	*Eleusine coracana* (L.) Gaertn.	321	7.2	66.82	1.92	11.18	2	NA	0.37	0.17	4.6	2.5	364	35	210
2	Bodi^*^	Cow pea, white	*Vigna unguiculata* (L.) Walp.^€^	320	21.3	53.77	1.14	11.7	8	NA	0.33	0.09	5.0	3.6	84.1	249	378
3	*Sutri*^$^	Rice bean	*Vigna umbellata* (Thumb.) Ohwi & H. Ohashi^€^	332	21.5	60.9	0.3	NA	NA	NA	NA	NA	NA	NA	302	NA	297
4	*Baturi dal*^¥^	Munmuna	*Vicia hirsuta* (L.) Gray	341	27	55.54	ND	8.79	178	12.45	0.22	1.15	17.1	3.8	77.5	ND	83.3
5	*Kantha ara*^#^	Kantha leaves	*Dentella repens* (L.) J.R. Forst. & G. Forst.	46	3.5	8	NA	7.1	11680	9	3.07	NA	81.1	1.0	425	7	NA
6	*Lupu ara*^∧^	Chhaya/ Kapurijari/ Gorakhbuti	*Aerva lanata L*. Juss. Ex Schult.	56	4.6	9.5	ND	5.9	21760	12	ND	ND	22.1	0.7	202	41	ND
7	*Kecho ara*^¥^	Punarnava	*Boerhavia procumbens* Banks ex Roxb.^€^	52	4.37	8.67	ND	2.47	2257	0.7	0.42	1.31	9.4	1.7	877.9	0.4	8.7
8	*Burju Baha*^¥^	Kachnar flower	*Bauhinia variegata* L.	83	2.98	17.7	ND	8.49	416	2.4	ND	0.3	3.4	0.7	404.9	ND	447.7
9	Adel sanga^*^	Tapioca	*Manihot esculenta* Crantz.	80	1.03	17.81	0.2	4.61	NA	17.6	0.07	0.02	0.8	0.1	25.9	26	42.6
10	*Haseaar sanga*^¥^	*-*	*Dioscorea quartiniana* A. Rich.^€^	72	4.4	13.49	ND	1.34	ND	3.1	1.09	ND	55.9	0.6	33.2	ND	26.4
11	*Soso*^$^	Marking nut (kernel)/ Bhelwa	*Semecarpus anacardium* L.f.	587	26.4	28.4	36.4	NA	NA	NA	NA	NA	6.1	NA	295	NA	NA
12	*Baadi*^$^	Banyan fruit	*Ficus benghalensis* L.	72	1.7	11.8	2	NA	NA	NA	NA	NA	NA	NA	364	NA	NA
13	*Redhayi/ Mangri*^$^	Walking catfish	*Clarias batrachus*	86	15	4.2	1	NA	NA	NA	NA	NA	0.7	NA	210	NA	290

*Text in Italics represents Mundari names*.

NA, Not available; ND, Not detected;

¥ *Laboratory analyzed; ^a^Retinol expressed in μg/100g for animal foods*.

Secondary data on nutrient analysis:

* (43);

∧ (45);

# (23);

$ *(44)*.

β Scientific name cited from secondary sources (28, 30, 40–45) and

€ *additionally verified from (46, 47)*.

** *All the foods listed in the table have at least one nutrient content per serve between 10 and 19% or >20% of RDA for Indian adults (49–51)*.

### Qualitative Enquires to Assess Factors Affecting IFs Consumption

**Figure 6:**
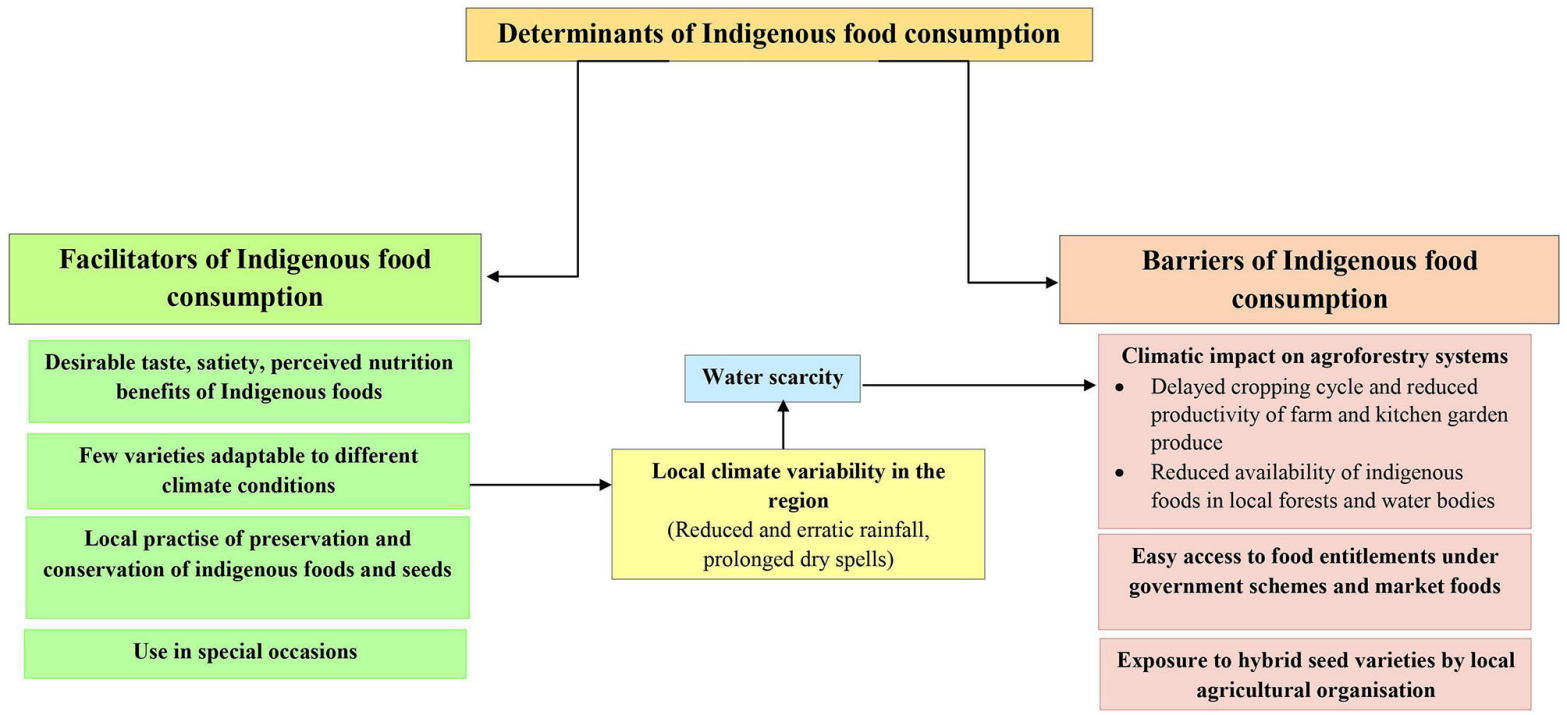
Factors influencing indigenous food consumption in Munda tribal community of Jharkhand, India.

A large number of IFs (*n* = 194) were reported during the free listing exercise, however, more than 50% of these foods were found to be either little used or historically consumed. Based on the themes identified from the qualitative enquires, a conceptual framework was developed which revealed a list of factors that directly or indirectly influenced the IF consumption in the community (Figure 6). The factors, which promoted the IF consumption were classified as facilitators, while those factors that hindered the consumption of IFs were classified as barriers.

#### Facilitators of Indigenous Food Consumption in Munda Tribal Community

The FGDs revealed four key themes that facilitate the consumption of IFs, which included, (i) desirable taste, satiety and perceived nutrition benefits of IFs, (ii) adaptability and resilience to climatic variability resulting in improved productivity and availability, (iii) the traditional practices of IF preservation and conservation which promote their incorporation in daily diets, and (iv) the cultural importance associated with IFs which facilitates their use in special occasions.

##### Desirable Organoleptic Properties and Perceived Nutritional Benefits of IFs

One of the main facilitators of IF consumption is the associated desirable taste and satiety giving properties of these foods. As one respondent said: “*Indigenous foods taste better and have more nutrition than hybrid varieties”* (Respondent number 5, male, study village 3, Torpa block, 24th June, 2019). Many indigenous varieties of rice (such as *Laldhan, Karnidhan, Reici baba, Karangadhan)*, GLVs (Koinaar leaves (“*Sing ara”: Bauhinia purpurea)*, Amaranth leaves (“*Leped ara”*: *Amaranth spinosus)*, Bengal gram leaves (“*Boot ara”*: *Cicer arietinum)*, Water spinach (“*Kalmi ara”*: *Ipomoea aquatica)* etc.) and flesh foods (like Puti fish (“*Pothi”*: *Barbus* spp.), Bele fish (“*Bale”*: *Glossogobius giuris)*, Spotted snakehead fish (“*Chodha Machli”*: *Channa punctatus)*, Snails (“*Loa suti”: Pila globosa*), Mussels (“*Setua”*: *Margaritifera margaritifera)*, Wild pig (“*Jungli suar”: Sus scrofa)*, Wild hen *(“Jungli murgi”: Galloanseres* sp*)* etc.) are locally known for their relishing flavor and are frequently consumed by the community members. For instance, one respondent stated*: “We grow indigenous rice varieties that taste good. If we eat indigenous food, we feel full for longer time duration*” (Respondent number 2, male, study village 4, Murhu block, 22nd June, 2019).

In addition to their desirable organoleptic properties, the community perceived the IFs to be nutritious, especially in comparison to hybrid varieties of crops. Certain varieties of indigenous rice (such as *Laldhan* and *Karnidhan*) and GLVs (Amaranth leaves/*Leped ara*) are particularly perceived as a good source of nutrition. As shared by respondents: “*We get lot of strength when we consume indigenous foods and they help us to improve the blood production in our body*” (Respondent number 1, male, study village 1, Torpa block, 21st June, 2019).

##### Resilience of IFs Amidst Climatic Variability

Apart from having desirable flavor and nutritional characteristics, the IFs are also perceived to be adaptable to changing climate conditions, topography and soil. The community reported the cultivation and consumption of different IFs that provide assured yield, irrespective of the climate situations. These included drought-resistant, indigenous rice varieties (*Laldhan, Reicibaba, Mansoridhan, Gitil baba, Dhusridhan*, and *Karangadhan*), which require less water. Certain indigenous varieties of pulses, such as Black gram (“*Rambada/Urad”: Vigna mungo*) and Horse gram (*Kulthi*), are reported to be cost-effective as their cultivation require minimal manpower (labor) or supplementary inputs (chemical fertilizers). For these reasons, these pulse crops are grown by almost every household in the community and are frequently incorporated in the main meals. The seeds of some indigenous rice and pulses are also reported to be insect-resistant. The community also reported accessing certain IFs that are available in forests and surrounding areas amidst adverse climatic conditions. These include specific varieties of indigenous roots and tubers like *Jat sanga* and GLVs like Amaranth leaves (*Leped ara)*, Katai leaves (“*Sarla ara”*: *Meyna pubescens*), Mata leaves (“*Mattha ara”*: *Antidesm aacidum*), Phutkal leaves (“*Phutkal ara”*: *Ficus virens)*, Pot Cassia *(“Chakod ara”: Senna obtusifolia)*, Garkha (“*Sirgiti ara”: Celosia argentea) and* Hurhura *(“Charmani ara”: Cleome monophylla)*.

##### Local Practice of Food and Seed Preservation and Conservation Within the Community

Due to the desirable taste and perceived nutritional benefits of IFs, the community reported a common practice of preservation and conservation of these foods. This local practice mainly involves the preservation of flavorsome varieties of GLVs, fruits and vegetables that have limited seasonal availability throughout the year. In case of GLVs, various indigenous varieties such as Phutkal leaves (*Phutkal ara*), Koinaar leaves (*Sing ara*), Beng leaves (*Chokke ara)*, Sweet potato leaves (“*Sanga ara”*: *Ipomoea batatas*), Garlic leaves (“*Lehsun ara”: Allium sativum*) and many others, are preserved using traditional methods of sun-drying. These sun-dried GLVs are reconstituted and cooked with rice water (*Maad*) and eaten as a curry along with rice. On the other hand, indigenous fruits like “*Dahu” (Artocarpus lakoocha)*, Ambada *(“Amda*”: *Spondias pinnata*) and Zizyphus (*Godaari*) are preserved using pickling methods. These fruit pickles are usually consumed alongside main meals, to add flavor.

In addition to IF preservation, the community also preserves the indigenous seeds of rice and pulses through a sun-drying technique. These sun-dried seeds are often wrapped in *Neem* leaves and stored in sacks till their use in the next sowing cycle.

##### Cultural Practices of Using IFs in Festivals and Special Occasions

Another factor that promotes the inclusion of IFs is their cultural value and importance among the Munda community. Mundas are intricately connected to their local cultures and traditions, some of which involve the use and consumption of different varieties of IFs during festivals and special occasions. For example, in the local festival of “*Sohrai*,” the community consumes various rice-based delicacies that are prepared using indigenous rice varieties. During special occasions like “*Annaprashan*,” [celebrated to mark the initiation of complementary feeding when a baby turns 6 months old) the baby is usually served a local dish “*Khichdi*,” which is prepared using indigenous rice, black gram (*Rambada/Urad)*, along with chopped leafy vegetables such as Koinaar leaves (*Sing ara*) and Amaranth leaves (*Leped ara*)]. Apart from these occasions, a local community tradition called “*Puwal*” (practiced after rice harvesting) also promotes the IF consumption. During “*Puwal*,” the entire village hosts a feast in which, mainly indigenous rice, pulses, vegetables and animal meats are served and consumed.

#### Barriers to Indigenous Food Consumption

Despite several facilitators associated with IFs consumption, a large number of IFs (55%) were reportedly under-utilized in the community. Based on our conceptual framework, we identified three main barriers toward the consumption of IFs. These include: (i) local climatic impact on agroforestry systems leading to reduced IF production, availability and consumption, (ii) easy access to foods purchased from local markets and/or distributed under government food security programmes, and (iii) promotion of high-yielding hybrid varieties by local agricultural organizations for food security.

##### Local Manifestation of Climate Variability on Agroforestry Leading to Poor IF Production, Access and Consumption

Local climate variability emerged as one of the main barriers for IF production and consumption. The Munda community reported erratic rainfall pattern, which includes a short rainy season followed by long periods of dry season. These climate-change induced events have significantly influenced the farming patterns of the community, which is mostly rain-fed. Due to low rainfall, water scarcity has become a major crisis in the region, leading to acute water shortage for crop irrigation. This has resulted in delayed farming cycles along with reduced productivity of both farm and kitchen garden produce. As shared by one respondent: “*Our farming is totally dependent on rain water. If rainfall is sufficient, then our farm produce is sufficient. If rainfall is inadequate, then our farming suffers*” (Respondent number 2, male, study village 1, Torpa block, 21st June, 2019).

Water scarcity has also impacted the crop diversity, with many people switching from multi-cropping to mono-cropping pattern: “*Earlier we used to cultivate a lot of crops in our farms and kitchen gardens, but now we cultivate less crops because of water shortage “*(Respondent number 4, male, study village 1, Torpa block, 21st June, 2019). Since the last two decades, the community has been mainly engaged in paddy cultivation, in contrast to earlier times, when different varieties of indigenous crops such as millets, GLVs and vegetables were cultivated in farms and kitchen gardens. One respondent stated: “*Due to less rainfall, we are not able to cultivate crops at the right time. Last year, due to shortage of water for crop irrigation, we couldn't grow many crops on our farm lands. The yield of the cultivated crop (paddy) was also very less”* (Respondent number 1, female, study village 4, Murhu block, 22nd June, 2019).

The local forests, that were once home to rich vegetation and biodiversity, have also been affected. The reduced and infrequent patterns of rainfall are gradually leading to forest degradation, because of which, there is reportedly a declining availability of several wild foods in the region. Some of the foods which are not consumed any more due to the loss of biodiversity include indigenous varieties of fruits such as Banyan (*Baadi*), Piar (“*Tarom”: Buchanania lanazan), “Loa” (Ficus racemosa)*, Bhui-gular *(“Aanri”*: *Ficus semicordata*), vegetables like Kachnar flower (*Burju Baha)*, Jirhul (“*Hutarba”: Indigofera cassioides*), GLVs like Hirmicha leaves (“*Muchdi ara”*: *Enhydra fluctuans)*, Karchul leaves (“*Lundi ara”*: *Butomopsis latifolia)*, Kantha leaves (*Kantha ara)*, Punarnava (*Kecho ara)* etc., roots and tubers like Tapioca (*Adel sanga), Hasaer sanga, Koolerumpa, Maisaranga* (classification NA), mushrooms like *Patkaud, Kurthiud, Bunumud* (classification NA) etc. and wild birds and animals like Porcupine (“*Jikki”:Erethizon dorsatum)*, Spotted Dove *(“Putam”:Streptopelia chinensis), Duhur, Sursuri and Askal* (classification NA). The local water bodies have mostly dried up, resulting in poor availability of indigenous fishes such as Gangetic mud eel (“*Noya”: Monopterus cuchia), Binghayi* and *Linda* (classification NA). All these factors have thus markedly reduced the IF production and access within the community, collectively leading to reduced consumption and use of diverse IFs in the local diets. For instance, one respondent said: “*Now, since it rains less, the crop production is less. Due to this, the availability of food decreases at home”* (Respondent number 3, male, study village 2, Murhu block, 20th June, 2019). While another respondent said: “*We eat very less amount of local (indigenous) pulses and green leafy vegetables. Now everyone eats potato along with rice, Because of this, the situation of food consumption is becoming worse”* (Respondent number 1, female, study village 4, Murhu block, 22nd June, 2019).

##### Easy Access to Non-indigenous Foods From Markets and Food Security Schemes

Due to climate impacts on agroforestry, many community members are unable to utilize the farm and forest produce for household consumption as well as income generation. In view of this, the community has started adopting alternative sources of livelihood such as wage laboring, working in factories, shops, hotels etc. The income generated from these jobs is mostly utilized for purchasing foods from local markets, which mainly comprise of non-indigenous pulses (green gram and lentils), vegetables (brinjal, cabbage, cauliflower, tomato, onion), GLVs (spinach and bathua leaves), and roots and tubers (potato). Moreover, the tribal community has access to food distributed under PDS, which supplies them with non-indigenous rice (and other food commodities such as wheat, sugar and salt) at highly subsidized rates. With diminished access to forest foods and increasing dependence on foods procured from markets and food security schemes, many people are failing to include IFs in their daily food basket. As one respondent said: “*Since the farming yield has reduced, we need to do labor work to earn money so that we can buy food from market and eat”* (Respondent number 2, male, study village 2, Torpa block, 23rd June, 2019).

##### Increasing Exposure to Hybrid Crop Varieties

The low crop yields associated with indigenous seeds and increased emphasis over modern farming methods by the local agricultural organizations, have led to changes in the traditional subsistence farming practices of Munda community. As a consequence, local farmers are utilizing the high-yielding hybrid seeds and chemical fertilizers for better farm productivity. For instance, one respondent said*: “…. Mainly hybrid paddy is being cultivated as compared to indigenous varieties because it gives twice or thrice more yields than indigenous varieties”* (Respondent number 2, male, study village 2, Murhu block, 20th June, 2019).

Since hybrid seeds provide better yield than indigenous seeds, the production of many indigenous crops have drastically reduced and/or ceased. For example, certain indigenous varieties of rice such as *Raajdhan, Daanidhan, Kanaaudhan, Raasdhan, Minjri, Jolpo baba, Dondo baba, Chorayagoda, Hengdahgoda, Hathipanjardhan, Jhilli baba, Heselsar, and Pasoda baba*, pulses like Munmuna (*Baturi Dal*) and millets like Finger millet (*Kodde*), Pearl millet (*Gaangi*), Little millet (*Gondli*), Sorghum (*Jowar*) which are known for their nutritional benefits, are no longer cultivated and consumed by the community, due to their reportedly low yield in comparison to hybrid crop varieties. All these factors are contributing to declining IF production, which further translates into their reduced consumption within the community. As shared by one of the respondents: “*….Yes, there is change in our consumption patterns. Earlier we used to eat local (indigenous) grains, but now we eat hybrid grains”* (Respondent number 5, male, study village 4, Torpa block, 22nd June, 2019).

## Discussion

TEK regarding a diverse range of IFs was observed within the Munda tribal community. These included several varieties of indigenous rice, GLVs, flesh foods, mushrooms, fruits, other vegetables, roots and tubers and pulses. The community accessed these IFs mostly from the cultivated and wild food environments i.e., farmlands, kitchen gardens, open fields, roadsides, wastelands, local water bodies, and forests. Despite awareness about several IFs, less than half of these were routinely consumed. Nonetheless, several of the routinely consumed as well as little used foods were found to be rich sources of micronutrients. Enquiries on factors favoring the consumption of IFs and probable barriers revealed specific socio-cultural and environmental factors.

Presently, rice is predominantly cultivated and consumed as a staple in the villages inhabited by Mundas. The community however, reported the historical cultivation and consumption of pearl millet, finger millet, little millet, and sorghum in the region. A similar trend on consumption of limited variety of cereals and diminishing consumption of diverse coarse cereals is observed in other tribal communities of Jharkhand as well as across India (23, 40, 57). Loss of coarse cereals like millets from the habitual Indian diet have been reported to have substantially reduced iron intake in the population, particularly in states where rice has replaced coarse cereals (58). Though our study community continued to grow several varieties of indigenous rice, hybrid varieties contributed to a major part of their produce as well as consumption. This practice of cultivation of high-yielding hybrid varieties of rice in this tribal community is consistent with the overall pattern of paddy cultivation among tribal groups, and, in general, in the state of Jharkhand. The available data from the state shows that only around 13 % of the total production of rice is contributed by traditional indigenous varieties (59). In this context, it is important to recognize that traditional pure lines are required to be preserved even for developing hybrid varieties with desirable traits. Due to the agricultural modernization throughout the country, the state of Jharkhand is also witnessing a fast decline of traditional varieties, which may have implications on the traditional cultivation systems of tribal communities (including Mundas), leading to the erosion of rich genetic diversity as well as the ancestral knowledge of preserving the seeds of traditional varieties (23, 57, 60–62). This may also compromise the socio-ecological resilience of indigenous communities (20). Further, studies from this region (including the present study) have also documented higher nutritional value of these indigenous rice varieties (63–65). Thus, there is an urgent need to conserve the indigenous varieties that are still cultivated, albeit, in lesser proportion of lands, by this tribal community. This community needs to be supported and empowered with knowledge and technology to promote and revive the cultivation of indigenous varieties with enhanced and assured yields.

Apart from cereals, the community also reported routine consumption of indigenous GLVs, roots and tubers, pulses and/or flesh foods; similar dietary patterns have been observed in other tribal communities of Jharkhand (40, 45, 57, 66). Many of the IFs reported in the study were found to be rich sources of proteins, vitamins and minerals especially iron, calcium, vitamin A, vitamin C and folic acid. The rich nutrient content of IFs consumed by Indian tribal communities have also been documented in other studies (23, 40, 45, 67, 68). A study on IFs of the Munda tribal community has documented consumption of some of the nutrient rich IFs in their routine diets. These included GLVs like Amaranth (*Amaranthus* spp.), Malabar spinach (*Basella alba*), Kangkong (*Ipomoea aquatica*), and Chenopodium (*Chenopodium album*), Moringa (*Moringa oleifera*), *Bauhinia* spp. and *Hibiscus sabdariffa* (69) which were also identified in the present study.

Despite a rich TEK of several IFs sourced from the natural food environment, a large proportion of these were infrequently used or historically consumed by the Mundas. Similar paradoxical findings on awareness about IFs and yet their poor consumption have been reported in studies conducted on Munda as well as other tribal communities of Jharkhand (23, 30, 57, 60).

The informal food literacy acquired from traditional knowledge associated with food, including agro-ecological knowledge (where and what type of food is produced), cultivation and production knowledge (how food is produced), and processing and consumption knowledge (how food is prepared and distributed) derived informally from people's everyday practices in home and community environments (70) can play a vital role in the maintenance and revival of traditional food systems. The Munda community was aware of the superior taste, nutritional, cultural, and agro ecological attributes of their IFs. Therefore, it is crucial to systematically explore and document these factors, and reinforce the informal food literacy with structured curriculum-based formal learning environments, which can facilitate value addition and resulting production and consumption of these foods by the coming generations. The community was aware of the climate resilient indigenous varieties of crops that they were cultivating. Literature suggests that indigenous farmers and local people perceive the impacts of climate change in their own ways and prepare for it through various adaptation practices (71). It is well documented that the adaptation of modern day agriculture to climate change would depend on the conservation and introduction of crop's wild relatives from the rich “native”/indigenous bio-diverse stocks that are managed by these indigenous people globally (72). In India, many indigenous communities, e.g., the Kondhs (inhabiting the forest villages in Koraput, Rayagada, Kandhamal, and Kalahandi districts of Odisha), who are unaware about the scientific premise of indigenous farming methods for their invaluable contribution in creating a climate resilient food systems, are effectively resisting climate change and maintaining the quality of their soil while protecting biodiversity in their local regions (73). In Arunachal Pradesh, the Adi tribe accesses several indigenous plant and animal species from diverse ecosystems, based on their sound knowledge of local biodiversity, and apply traditional agronomic, cultural, and harvest strategies to conserve and sustain their natural resources against abrupt weather anomalies (20, 74–78).

The changing agrarian practice of mono-cropping pattern along with diminished cultivation and production of indigenous cereals owing to water scarcity that reportedly resulted from impacts of local climate change in the region was a barrier to IF consumption. The climate change has also affected the availability of wild foods from forests and other natural environments. The declining availability of IFs is also being observed in other regions of Jharkhand (79, 80), which is worrisome from both nutritional and ecological point of view. Promotion of high yield varieties of paddy by agricultural organizations and reliance on non-indigenous commercial foods available in open market and mono-diets distributed under the government food security program were further identified as other barriers. Studies have documented the impact of climate uncertainties on indigenous farming practices that go beyond reductions in yield and influence how farmers make choices about the timing of planting, soil management, and the use and spatial distribution of narrow range of crop varieties (81, 82). Furthermore, household processing techniques to preserve and detoxify native foods rely on key environmental and climatic resources, which may be vulnerable to climatic shifts (81). Studies have also documented the role of agricultural organizations in limiting IF production as well as consumption (80, 83). Apart from changing agricultural production, it is well documented that improved access to market has led to consumption of non-indigenous market foods (usually rich in sugar, fat, and salt) as part of the daily diets of tribal communities in Jharkhand (84). The shift from consumption of nutrient-dense IFs to energy dense foods is indicative of the ongoing nutrition transition, a trend that has also been reported among other indigenous communities of India as well as globally (85–87). Changing preferences toward consumption of market foods, overdependence on PDS, along with shift toward mono-cropping patterns, may not only result in reduced consumption of nutrient dense IFs, but may also collectively impact the local, resilient food systems of these communities.

## Study Limitations

Although we analyzed and compiled nutritive values for 102 IFs of Munda community, owing to limitations around seasonal availability and accessibility of some foods, taxonomic classification could not be performed for all IFs, as a result of which, their nutritive values could not be documented. Moreover, though several IFs were found to be rich sources of nutrients, it is important to assess the anti-nutritional components and toxicity levels in these foods. Nonetheless, these gaps can be addressed by conducting future research in the study community.

## Conclusion

The Munda community reported a diverse food system, demonstrated TEK about several IFs, yet had underutilization of IFs in their daily diets. The perceived socio-cultural value assigned to IFs was an important facilitator to their consumption while environmental factors like climatic shifts and the resulting influences on the agroforestry systems emerged as potential barriers. The food systems of Munda tribes can potentially contribute to biodiversity conservation with low energy inputs and climate change mitigation. This may prove to be a time tested model contributing to maintenance and propagation of modern sustainable agriculture systems, which may be key to achieving SDG 2 targets of eliminating hunger and malnutrition and improving sustainability of food systems (6). Relevant measures are required to gear the transformation toward more localized, nutritious and climate-resilient food and production systems. Supportive policies and the grassroots developmental and agricultural extension initiatives can play an instrumental role in ensuring improved IF production, access and utilization, through a range of activities. Nutrient-rich IFs need to be incorporated in government-run supplementary nutrition programmes (MDM, ICDS, and PDS) for increasing IF consumption, which will not only address the nutritional vulnerability of the tribal populations, but will also create more demand for locally produced foods. Creation of community seed banks for indigenous seeds distribution, education on sustainable farming methods for conservation of local landraces as well as drought-resistant varieties, supporting communities for establishing home gardens and conducting nutrition education sessions that reinforce TEK and raise interest about IFs and their nutritional significance should be encouraged (7, 78). All these actions are necessary elements for preserving resilient, nutritious and sustainable food systems.

In conclusion, our findings indicate the importance of retaining and reinforcing TEK and informal food literacy about IFs among Munda community while promoting and supporting climate resilient attributes of their IF systems.

## Data Availability Statement

The original contributions presented in the study are included in the article/Supplementary Material, further inquiries can be directed to the corresponding author/s.

## Ethics Statement

All procedures involving humans in this study were approved by the Institutional Ethics Committee at Indian Institute of Public Health-Delhi, Public Health Foundation of India and All India Institute of Medical Sciences, New Delhi. Administrative approvals from authorities at district level was also taken. Written informed consent was obtained from all participants who were literate. Third-party witnessed verbal consents were obtained from illiterate participants.

## Author Contributions

SG-J and AS conceived and designed the study with overall supervision from JF. SG-J, RK, SB, and AS supervised the entire data collection process. GS, RK, and SB did the data analysis. SG-J, RK, and SB prepared the first draft of the manuscript. AS, GS, SD, and JF critiqued and modified the draft. SG-J had final responsibility for the decision to submit for publication. All authors read and approved the final version.

## Conflict of Interest

The authors declare that the research was conducted in the absence of any commercial or financial relationships that could be construed as a potential conflict of interest.
