# Supplementary material for: Traditional Food Environment and Factors Affecting Indigenous Food Consumption in Munda Tribal Community of Jharkhand, India
Source: Front Nutr. 2021 Feb 1;7:600470. doi: 10.3389/fnut.2020.600470 (PMC7882711; doi:10.3389/fnut.2020.600470)
Supplement: Supplementary file 1 [file Table_1.docx]

| **S. No** | **Test Parameter/Standard** | **Methodology** | **Reference method** | **Limit of Quantification (LOQ)** |
| --- | --- | --- | --- | --- |
| 1. | Energy (Kcal/100gm) | Gravimetric method | IS:9487-1980(RA-2005) | 1.0 |
| 2. | Protein (g/100gm) | Titrimetric method | FSSAI Lab Manual | 0.1 |
| 3. | Total Fat (g/100gm) | Gravimetric method | IS:9487-1980(RA-2005) | 0.02 |
| 4. | Total Carbohydrate (g/100gm) | By difference | IS:1656-2007 | 0.1 |
| 5. | Dietary Fibre (g/100gm) | Enzymatic Gravimetric method | AOAC 20^th^ edition 985.29 | 0.5 |
| 6. | Vitamin A (as β- carotene) (µg/100gm) | High Performance Liquid Chromatography (HPLC) | QA.16.5.163 | 5.0 |
| 7. | Vitamin B_1_,B_2_( mg/100gm) | High Performance Liquid Chromatography (HPLC) | QA.16.5.9 | 0.2 |
| 8. | Vitamin C (mg/100gm | High Performance Liquid Chromatography (HPLC) | IS:5838-1970 (RA-2005) | 1.0 |
| 9. | Folic acid (µg /100gm) | High Performance Liquid Chromatography (HPLC) | QA.16.5.9 | 0.1 |
| 10. | Calcium, Iron, Zinc (mg/100gm) | Inductively Coupled Plasma Mass Spectrometry (ICP-MS) | QA.16.5.2/AOAC 20^th^ edition | 0.05 |

**Supplementary Table 1: List of parameters and relevant methodological details for nutrient analysis**
