# Supplementary material for: Traditional Food Environment and Factors Affecting Indigenous Food Consumption in Munda Tribal Community of Jharkhand, India
Source: Front Nutr. 2021 Feb 1;7:600470. doi: 10.3389/fnut.2020.600470 (PMC7882711; doi:10.3389/fnut.2020.600470)
Supplement: Supplementary file 2 [file Table_2.docx]

**Supplementary Table 2:**  Indigenous foods with taxonomic classification, part(s) consumed, place of procurement, preference and seasonality

| **S No.** | **Local (Mundari) Name** | **Common name** | | **Scientific Classification** | **^β^Scientific Name** | **Part Consumed** | **Accessed/**  **Grown** | **Preference (Commonly consumed/Little used/Historically consumed)** | **Season (Summer/**  **Monsoon /Winter /All seasons)** |
| --- | --- | --- | --- | --- | --- | --- | --- | --- | --- |
| 1-29. | *Laaldhan/Arababa^$^, Raajdhan^#^, Jedengdhan^$^, Daanidhan^#^, Pasodababa^#^, Karangadhan^$^, Kanaaudhan^#^, Raasdhan^#^,Minjri^#^, Aara/Aarodhan^#^, Jolpobaba^#^, Dondo baba^#^, Gitil Baba^$^, Pundigoda/Pundibaba^$^, Rieci baba^$^, Karnidhan^$^, Chorayagoda^#^, Hengdahgoda^#^, Safeddhan^$^, Panchanwayedhan^$^, Dusridhan^$^, Hathipanjardhan^#^, Mansori^$^, Anthanbey Dhan^#^, Jhillidhan^$^, Sambalpuri/Sangolpuri^$^, Heselsar^#^, Munadhan^$,^ Kalamdanidhan^#^* | Varieties of rice | | ✓ | *Oryza sativa* L. | Grain | Field | Commonly consumed*^$^*, Little used*^#^* | Monsoon (sowing) and Winter  (harvesting) |
|  | *Jondra/Makai* | Maize | | ✓ | *Zea mays* L. | Cob | Field | Commonly consumed | All season |
|  | *Gaangi* | Pearl millet | | ✓ | *Pennisetum glaucum* (L.) R.Br. | Kernel | Field | Little used | Winter |
|  | *Kodde* | Finger millet | | ✓ | *Eleusine coracana* (L.) Gaertn. | Grain | Field | Little used | Winter |
|  | *Jowar* | Sorghum | | ✓ | *Sorghum bicolor* (L.) Moench | Grain | Field | Little used | Winter |
|  | *Gondli* | Little millet | | ✓ | *Panicum miliare*Lam. | Grain | Field | Little used | Winter |
|  | *Rambada/Urad* | Black gram dal | | ✓ | *Vigna mungo* (L.) Hepper | Seed | Field | Commonly consumed | Winter |
|  | *Bodi* | Cow pea, white | | ✓ | *Vigna unguiculata* (L.) Walp. | Seed and vegetable | Field | Little used, Commonly consumed  (Vegetable) | ^a^ Monsoon and Winter |
|  | *Dangbodi* | Cow pea, brown | | ✓ | *Vigna unguiculata* (L.) Walp. | Seed | Field | Little used | ^a^ Monsoon and Winter |
|  | *Sutri* | Rice bean | | ✓ | *Vigna umbellata* (Thumb.) Ohwi & H. Ohashi | Seed | Field | Little used | Winter |
|  | *Kulthi* | Horse gram | | ✓ | *Macrotyloma uniflorum* (Lam.) Verdc. | Seed | Field | Commonly consumed | ^a^ Monsoon and Winter |
|  | *Khesari* | Khesari | | ✓ | *Lathyrus sativus* L. | Seeds and leaves | Field | Little used | Winter |
|  | *Baturi/Teeri riti* | Munmuna | | ✓ | *Vicia hirsuta* (L.) Gray | Seeds and leaves | Field | Little used | ^a^ Monsoon and Winter |
|  | *Sing ara* | Koinaar leaves | | ✓ | *Bauhinia purpurea* L. | Leaves | Forest and Wastelands | Commonly consumed | Summer |
|  | *Saru ara* | Colocasia leaves | | ✓ | *Colocasia esculenta* (L.) Schott | Leaves and root | Forest | Commonly consumed | ^a^ Monsoon and Winter |
|  | *Kantha ara* | Kantha leaves | | ✓ | *Dentella repens* (L.) J.R. Forst. & G. Forst. | Leaves | Field (wild) | Little used | Summer and Monsoon |
|  | *Dali/Dail ara* | Nunia leaves | | ✓ | *Portulaca quadrifida* L. | Leaves | Bakdi | Little used | ^a^ Monsoon and Winter |
|  | *Kaddu ara* | Bottle gourd leaves | | ✓ | *Lagenaria siceraria* (Molina) Standl. | Leaves | Bakdi | Little used | Summer |
|  | *Mattha ara* | Mata leaves | | ✓ | *Antidesma acidum* Retz. | Leaves | Field (wild) | Commonly consumed | Monsoon |
|  | *Sirgiti ara/ Siliary ara* | Garkha/ Gadrya/ Garke | | ✓ | *Celosia argentea* L*.* | Leaves | Forest | Commonly consumed | Monsoon |
|  | *Aloo ara* | Potato leaves | | ✓ | *Solanum tuberosum* L. | Leaves | Field and Bakdi | Little used | Winter |
|  | *Muri/Munga ara* | Drumstick leaves | | ✓ | *Moringa oleifera* Lam. | Leaves | Bakdi | Commonly consumed | All season |
|  | *Leped ara* | Amaranth leaves | | ✓ | *Amaranthus spinosus* L. | Leaves | Bakdi and Pastures | Commonly consumed | Monsoon |
|  | *Garundi ara* | Ponnaganni | | ✓ | *Alternanthera sessilis* (L.) R.Br. ex DC. | Leaves | Bakdi (wild) and Pastures | Commonly consumed | Summer |
|  | *Undku ara* | Kena leaves | | ✓ | *Commelina benghalensis* L. | Leaves | Bakdi (wild) and Pastures | Commonly consumed | Monsoon |
|  | *Khadia ara* | Dhurup leaves | | ✓ | *Leucas lavandulifolia* Sm. | Leaves |  | Little used | Monsoon |
|  | *Boot ara* | Bengal gram leaves | | ✓ | *Cicer arietinum* L. | Leaves | Bakdi | Commonly consumed | Winter |
|  | *Ohio ara* | - | | ✓ | *Trianthema portulacastrum* L. | Leaves | Forest | Little used | Autumn |
|  | *Hesa ara* | Banyan leaves | | ✓ | *Ficus benghalensis* L. | Leaves and fruit | Forest | Little used | Monsoon |
|  | *Sarla ara* | Katai leaves | | ✓ | *Meyna pubescens* (Kurz.) Robyns | Leaves | Forest | Commonly consumed | ^a^ Monsoon and Winter |
|  | *Susni ara/Chatom ara* | Sunsuni leaves | | ✓ | *Marsilea minuta* L*.* | Leaves | Wastelands and Pastures | Commonly consumed | All season |
|  | *Lupu ara* | Chhaya / Kapurijari / Gorakhbuti | | ✓ | *Aerva lanata* (L.) Juss. Ex Schult. | Leaves | Field (wild) | Little used | All season |
|  | *Epil ara/kudrum* | Gogu leaves, green | | ✓ | *Hibiscus sabdariffa* L. | Leaves and fruit | Bakdi and Field | Commonly consumed | Monsoon |
|  | *Chimti/Mui ara* | Chimti leaves | | ✓ | *Polygonum plebeium* R.Br. | Leaves | Field | Commonly consumed | Winter and Spring |
|  | *Charmani ara* | Hurhura | | ✓ | *Cleome monophylla* L. | Leaves | Field | Commonly consumed | Monsoon |
|  | *Lal bhaji/Lal saag* | Amaranth, tender, red leaves | | ✓ | *Amaranthus retroflexus* L. | Leaves | Bakdi and Market | Little used | ^a^ Monsoon and Winter |
|  | *Kecho ara* | Punarnava | | ✓ | *Boerhavia procumbens* Banks ex Roxb. | Leaves | Bakdi (wild) | Little used | Monsoon |
|  | *Lehsun ara* | Garlic leaves | | ✓ | *Allium sativum* L. | Leaves | Field | Little used | Winter |
|  | *Bir chhatom ara* | Khatti buti | | ✓ | *Oxalis corniculata* L. | Leaves | Bakdi and Pastures | Commonly consumed | Winter |
|  | *Uri le ara* | Purslane | | ✓ | *Portulaca oleracea* L. | Leaves | Field and Bakdi | Little used | Monsoon |
|  | *Kohna/Ketha* | Ash gourd leaves | | ✓ | *Benincasa hispida* (Thunb.) Cogn. | Leaves and vegetable | Bakdi | Commonly consumed, Little used(vegetable) | Summer |
|  | *Chakod ara* | Pot Cassia | | ✓ | *Senna obtusifolia* (L.) H.S. Irwin & Barneby | Leaves | Pastures | Commonly consumed | ^c^ Summer and Winter |
|  | *Kalmi ara* | Water spinach | | ✓ | *Ipomoea aquatica* Forssk. | Leaves | Marshy land | Commonly consumed | Monsoon |
|  | *Lundi ara* | Karchul leaves | | ✓ | *Butomopsis latifolia* (D.Don) Kunth. | Leaves | Pastures | Little used | Monsoon |
|  | *Uli ara* | Creeping marsh weed | | ✓ | *Limnophila repens* (Benth.) Benth. | Leaves | Field | Little used | Monsoon |
|  | *Sugu ara* | Sugga leaves | | ✓ | *Ophioglossum reticulatum* L. | Leaves | Field | Little used | Autumn |
|  | *Kotle ara* | Patsan | | ✓ | *Hibiscus cannabinus* L. | Leaves | Bakdi | Commonly consumed | Monsoon |
|  | *Soredhe/ Bir/ Rimil ara* | Dheniani | | ✓ | *Olax scandens* Roxb. | Leaves | Forest | Little used | Monsoon |
|  | *Chaari ara* | Kharika leaves | | ✓ | *Spergula pentandra* L. | Leaves | Field and Pastures | Little used | Winter |
|  | *Piring ara* | Netho saag | | ✓ | *Medicago denticulata* Willd. | Leaves | Forest | Little used | Monsoon |
|  | *Phutkal ara* | Phutkal leaves | | ✓ | *Ficus virens* Aiton | Leaves | Forest and Wastelands | Commonly consumed | Winter |
|  | *Chiringid ara* | Akra | | ✓ | *Vicia sativa* L. | Leaves | Bakdi | Commonly consumed | Winter |
|  | *Posta ara* | Poppy leaves | | ✓ | *Papaver somniferum* L. | Leaves | Bakdi | Little used | Winter |
|  | *Jojo ara* | Tamarind leaves | | ✓ | *Tamarindus indica* L. | Leaves | Forest and Roadsides | Commonly consumed | Monsoon |
|  | *Budilaie ara* | Khatta saag | | ✓ | *Cissus auriculata* Roxb. | Leaves | Field | Little used | Monsoon |
|  | *Kakaru ara* | Pumpkin leaves | | ✓ | *Cucurbita pepo* L. | Leaves | Bakdi | Commonly consumed | ^c^ Summer and Winter |
|  | *Sanga ara* | Sweet potato leaves | | ✓ | *Ipomoea batatas (*L.) Lam. | Leaves | Bakdi and Field | Commonly consumed | Monsoon |
|  | *Poi Saag* | Malabar spinach | | ✓ | *Basella alba* L. | Leaves | Bakdi | Commonly consumed | Autumn |
|  | *Kauwa ara* | Kauwa leaves | | ✓ | *Rungia quinqueangularis* Koen. | Leaves | Forest | Commonly consumed | Monsoon |
|  | *Beng saag/ Chokke ara* | Beng leaves | | ✓ | *Centella asiatica* (L.) Urb*.* | Leaves | Bakdi and Pastures | Commonly consumed | ^c^ Summer and Winter |
|  | *Tir ara/ Lochkor ara* | Arrow head | | ✓ | *Sagittaria latifolia* L. | Leaves | Marshy land | Little used | Monsoon |
|  | *Muchdi ara* | Hirmicha leaves | | ✓ | *Enhydra fluctuans* Lour. | Leaves | Field and market | Little used | ^a^ Monsoon and Winter |
|  | *Losodar ara* | Kuttra, Kapur | | ✓ | *Limnophila aromatica* (Lam.) Merr | Leaves | Bakdi | Little used | Winter |
|  | *Bhaduli ara* | Lasora | | ✓ | *Cordia macleodii* (Griff.) Hook. f. &Thomson | Leaves | Forest | Little used | Winter |
| 93-96. | *Budimikudi, Derang saag, Gedi ara, Heteteyo ara***** | Varieties of GLVs | | | | Leaves | Forest, Pastures  and Wastelands | Little used | ^b^ Monsoon to Winter |
|  | *Bir Karela* | Bitter gourd | | ✓ | *Momordica charantia* L. | Vegetable | Forest and Bakdi | Little used | Monsoon |
|  | *Manal/Simbi* | Field beans, tender | | ✓ | *Lablab purpureus* (L.) Sweet | Vegetable | Bakdi and Market | Commonly consumed | ^c^ Summer and Winter |
|  | *Jhinga/ Dodo/Doro* | Ridge gourd | | ✓ | *Luffa acutangula* (L.) Roxb. | Vegetable | Bakdi and Market | Commonly consumed | All season |
|  | *Kundri* | Kovai | | ✓ | *Coccinia grandis* (L.) Voigt | Vegetable | Bakdi | Commonly consumed | All season |
|  | *Jiri ba* | Sanai phool | | ✓ | *Crotalaria juncea* L. | Vegetable | Field | Commonly consumed | Winter |
|  | *Burju Baha* | Kachnar flower | | ✓ | *Bauhinia variegata* L. | Vegetable | Forest | Little used | Winter |
|  | *Hutarba* | Jirhul | | ✓ | *Indigofera cassioides* D.C. | Vegetable | Forest | Little used | Winter |
|  | *Kutumba / Hanjen* | Kutma | | ✓ | *Solanum torvum*  Swartz. | Vegetable | Bakdi and Wastelands | Commonly consumed | Monsoon |
|  | *Bans* | Bamboo tender | | ✓ | *Bambusa vulgaris* Schrad. ex J.C. Wendl. | Vegetable | Forest and Roadsides | Commonly consumed | Monsoon |
|  | *Haseaar sanga* | - | | ✓ | *Dioscorea quartiniana* A. Rich. | Root | Forest | Little used | ^a^ Monsoon and Winter |
|  | *Pindi* | Oal | | ✓ | *Amorphophallus paeoniifolius* (Dennst.) Nicolson | Root | Bakdi and Market | Commonly consumed | ^b^Monsoon to Winter |
|  | *Haranbho/Piski sanga* | Ban-aloo/ Gethia kanda | | ✓ | *Dioscorea bulbifera* L. | Root | Forest and Market | Commonly consumed | Monsoon |
|  | *Haatikata/Aaru/Jat sanga* | Khamaloo/ Chupri-aloo | | ✓ | *Dioscorea alata* L. | Root | Forest | Commonly consumed | Winter |
|  | *Adel sanga* | Tapioca | | ✓ | *Manihot esculenta* Crantz. | Root | Forest | Little used | Winter |
|  | *Toti* | Pechki | | ✓ | *Colocasia esculenta* (L.) Schott. | Root | Forest | Commonly consumed | Monsoon |
| 112-113. | *Koolerumpa, Maisarsanga***** | Varieties of roots and tubers | | | | Root | Forest | Little used | ^b^ Monsoon to Winter |
|  | *Amda* | Ambada | ✓ | | *Spondias pinnata* (L.f.) Kurz. | Fruit | Forest | Commonly consumed | Summer |
|  | *Mahua* | Mahua (ripe) | ✓ | | *Madhuca longifolia* (J. Koenig. ex L.) J.F. Macbr. | Fruit | Forest | Little used | Summer |
|  | *Soso* | Marking nut (kernel) / Bhelwa | ✓ | | *Semecarpus anacardium* L.f. | Fruit | Forest | Little used | Spring |
|  | *Godaari* | Zizyphus | ✓ | | *Zizyphus jujuba* Mill. | Fruit | Forest | Commonly consumed | Winter |
|  | *Tiril / Kendu* | Tumki | ✓ | | *Diospyros melanoxylon* Roxb. | Fruit | Forest | Commonly consumed | Monsoon |
|  | *Baru* | Kusum | ✓ | | *Schleichera* oleosa (Lour.) Merr. | Fruit | Forest | Commonly consumed | Summer |
|  | *Dahu* | Barhar | ✓ | | *Artocarpus lakoocha* Roxb. | Fruit | Forest | Commonly consumed | Summer |
|  | *Tarom/Char* | Char/ Piar | ✓ | | *Buchanania lanazan* Spreng. | Fruit | Forest | Little used | Spring |
|  | *Tamras* | Palmyra fruit, ripe | ✓ | | *Borassus flabellifer* L. | Fruit | Roadsides  and Wastelands | Commonly consumed | Monsoon |
|  | *Loa/Dumur* | Gular | ✓ | | *Ficus racemosa* L. | Fruit | Forest | Little used | Monsoon |
|  | *Sinju* | Wood apple | ✓ | | *Aegle marmelos* (L.) Correa | Fruit | Roadsides and Wastelands | Commonly consumed | Summer |
|  | *Dhela* | Akola/ Ankot | ✓ | | *Alangium salviifolium* (L.f.) Wangerin | Fruit | Forest | Little used | Monsoon |
|  | *Aanri* | Bhui-gular/ Khaina/ Khunia | ✓ | | *Ficus semicordata* Buch.-Ham. Ex Sm. | Fruit | Forest | Little used | Monsoon |
|  | *Rugra/Putuh* | Mushroom | ✓ | | *Geastrum* | Fruiting body of fungi | Forest | Commonly consumed | Monsoon |
|  | *Tumbaud* | Mushroom | ✓ | | *Lycoperdon* | Fruiting body of fungi | Forest | Commonly consumed | Monsoon |
|  | *Indiud* | Mushroom | ✓ | | *Termitomyces albuminosa* | Fruiting body of fungi | Forest | Commonly consumed | Monsoon |
|  | *Koodeud/ Kundaud* | Mushroom | ✓ | | *Termitomyces clypeatus* | Fruiting body of fungi | Forest | Commonly consumed | Monsoon |
|  | *Haathikataud* | Mushroom | ✓ | | *Boletus edulis* | Fruiting body of fungi | Forest | Commonly consumed | Monsoon |
|  | *Gendeud* | Mushroom | ✓ | | *Geastrum* spp. | Fruiting body of fungi | Forest | Little used | Monsoon |
| 133-148 | *Gitilud^$^, Pidhiud^$^, Koyaansakam^$^, Rampatka/Patka ud^$^, Simoodali/simdaliud/Sendaliud^$^, Badhayiud/Badheud^$^, Patdhaud^#^,Tormodaud/Bhorondaud^$^, Aataud^#^, Kurthiud^#^, Chokerotte^#^, Bengputu^#^, Gomaud^#^, Bedaud^#^, Bunumud^#^, Dasayeud^#^, Lundiud ^#^***** | Varieties of mushrooms | | | | Fruiting body of fungi | Forest | Commonly consumed*^$^,* Little used*^#^* | Monsoon |
|  | *Redhayi/Mangri* | Walking Catfish | ✓ | | *Clarias batrachus* | Meat | Water bodies | Little used | All season |
|  | *Pothi/ /Chirpi Machli* | Puti fish | ✓ | | *Barbus* sp. | Meat | Water bodies | Commonly consumed | Monsoon |
|  | *Bele Machli* | Bele fish | ✓ | | *Glossogobius giuris* | Meat | Water bodies | Commonly consumed | All season |
|  | *Koronjo/Tilapia* | Blue tilapia | ✓ | | *Oreochromis aureus* | Meat | Water bodies | Commonly consumed | All season |
|  | *Noya* | Gangetic mud eel | ✓ | | *Monopterus cuchia* | Meat | Water bodies | Little used | Monsoon |
|  | *Chudhako/Chodha machli* | Spotted snake head fish | ✓ | | *Channa punctatus* | Meat | Water bodies | Commonly consumed | Monsoon |
|  | *Aira* | - | ✓ | | *Clupea cultrate* | Meat | Water bodies | Little used | All season |
|  | *Sundi/Getu machli* | - | ✓ | | *Lepidocephalichthys guntea* | Meat | Water bodies | Commonly consumed | Monsoon |
|  | *Budu* | - | ✓ | | *Danio rerio* | Meat | Water bodies | Commonly consumed | Monsoon |
| 159-162. | *Maksakam^#^, Linda^#^, Kakandahayi^#^, Binghayi^#^***** | Varieties of fishes | | | | Meat | Water bodies | Little used | Monsoon |
|  | *Jikki / Saahil* | Porcupine | ✓ | | *Erethizon dorsatum* | Meat | Water bodies | Little used | Monsoon |
|  | *Loa suti* | Snail | ✓ | | *Pila globosa* | Meat | Water bodies | Commonly consumed | Monsoon |
|  | *Setua/Keyosuti* | Mussels | ✓ | | *Margaritifera margaritifera* | Meat | Water bodies | Commonly consumed | Monsoon |
|  | *Jangli murgi* | Wild hen | ✓ | | *Galloanseres* sp. | Meat | Forest | Commonly consumed | All season |
|  | *Hadhoga/Siyar* | Fox | ✓ | | *Vulpes vulpes* | Meat | Forest | Little used | All season |
|  | *Mussa/Guddu* | Field's rat | ✓ | | *Rattus argentiventer* | Meat | Forest | Commonly consumed | All season |
|  | *Bando* | Wild cat | ✓ | | *Felis catus* | Meat | Forest | Little used | All season |
|  | *Jungli suar* | Wild pig | ✓ | | *Sus scrofa* | Meat | Forest | Commonly consumed | All season |
|  | *Jungli mor* | Peacock | ✓ | | *Pavo cristatus* | Meat | Forest | Little used | All season |
|  | *Gilhari ka mass* | Squirrel meat | ✓ | | *Funambulus* sp. | Meat | Forest | Little used | All season |
|  | *Dhamna* | Indian Rat Snake | ✓ | | *Pytas mucosa* | Meat | Forest | Little used | All season |
|  | *Terom* | - | × | | *-* | Meat | Forest | Little used | All season |
|  | *Demta / Hau anda* | Eggs of red ants | ✓ | | *Oceophylla smaragdina* | Egg | Forest | Commonly consumed | All season |
|  | *Kabutar* | Pigeon | ✓ | | *Columba livia domestica* | Meat | Forest | Commonly consumed | All season |
|  | *Maina* | Indian myna | ✓ | | *Acridotheres tristis* | Meat | Forest | Little used | All season |
|  | *Chitri* | Grey Partridge | ✓ | | *Francolinus pondicerianus* | Meat | Forest | Little used | All season |
|  | *Ghaghar* | Common Quail | ✓ | | *Coturnix coturnix* | Meat | Forest | Little used | All season |
|  | *Putam* | Spotted Dove | ✓ | | *Streptopelia chinensis* | Meat | Forest | Little used | All season |
|  | *Burdulu/Ufia* | Winged termites | ✓ | | *Coptotermes* spp. | Meat | Forest | Little used | All season |
| 182-184 | *Duhur, Sursuri, Askal***** | Varieties of birds | | | | Meat | Forest | Little used | All season |
| 185 | *Neelirasi* | Indian forest honey bee | ✓ | | *Apis cerana indica/ Apis dorsata* | Larva | Forest | Little used | All season |
| 186 | *Tumbuli* | Hornet & Wasp | ✓ | | *Vespa* spp. / *Ropalidia* spp. | Larva | Forest | Little used | All season |
| 187 | *Madumakkhi* | Bees | ✓ | | *‎Apis mellifera* | Honey | Forest | Little used | All season |

***Note*:** Text in Italics represents Mundari name

**** Taxonomic classification not available

^$^Commonly consumed

*^#^* Little used

**^β^** Scientific name cited from secondary literature: Singh and Kumar, 2016 (22); Singh and Kumar, 2014 (24); Singh and Kumar, 2015 (35); Singh LR, Rani V. 2019 (36); Longvah et al., 2017 (37 ); Gopalan et al., 1989 (38) ; Ghosh-Jerath et al., 2020 (39); Ghosh-Jerath et al.,2015 (42); “Tropicos - Home,” 1982 (40) and “Home — The Plant List,” 2002 (41)

^a^ Monsoon and Winter- Available in monsoon as well as in winter season

^b^ Monsoon to Winter - Available in monsoon, autumn and winter season

^c^ Summer and Winter- Available in summer as well as in winter season
