# Supplementary material for: Traditional Food Environment and Factors Affecting Indigenous Food Consumption in Munda Tribal Community of Jharkhand, India
Source: Front Nutr. 2021 Feb 1;7:600470. doi: 10.3389/fnut.2020.600470 (PMC7882711; doi:10.3389/fnut.2020.600470)
Supplement: Supplementary file 3 [file Table_3.docx]

**Supplementary Table 3: Nutritive value of indigenous foods based on laboratory analysis and secondary data**

| **S. No.** | **Food item (Mundari Name)** | **Common name (English/ Hindi)** | **Scientific name**^β^ | **Energy (Kcal/ 100g)** | **Protein (g/ 100g)** | **Carbohydrate (g/ 100g)** | **Fat (g/ 100g)** | **Dietary fibre (g/ 100g)** | **β- Carotene/Retinol(µg/ 100g)** | **Vit C(mg/ 100g)** | **Vit B1 (mg/100g)** | **Vit B2 (mg/100g)** | **Total**  **folate (µg/ 100g)** | **Iron (mg/100g)** | **Zinc (mg/ 100g)** | **Calcium (mg/ 100g)** | **Phosphorus (mg/ 100gm)** |
| --- | --- | --- | --- | --- | --- | --- | --- | --- | --- | --- | --- | --- | --- | --- | --- | --- | --- |
|  | *Pundigoda* ^¥^ | Rice | *Oryza sativa* L. | 351 | 4.28 | 80.7 | 1.26 | 5.19 | ND | ND | 0.44 | 0.12 | 3 | 0.4 | 0.1 | 20.6 | 37.6 |
|  | *Jondra/ Makai** | Maize | *Zea mays* L. | 120 | 3.6 | 22.69 | 1.4 | 3.67 | 36 | NA | 0.17 | 0.12 | 63 | 0.71 | 0.97 | 6.4 | 163 |
|  | *Gaangi** | Pearl millet | *Pennisetum glaucum* (L.) R.Br^€^ | 348 | 11 | 61.78 | 5.43 | 11.49 | NA | NA | 0.25 | 0.2 | 36 | 6.4 | 2.8 | 27.3 | 289 |
|  | *Kodde* * | Finger millet | *Eleusine coracana* (L.) Gaertn. | 321 | 7.2 | 66.82 | 1.92 | 11.18 | 2 | NA | 0.37 | 0.17 | 35 | 4.6 | 2.5 | 364 | 210 |
|  | *Jowar** | Sorghum | *Sorghum bicolor* (L.) Moench. ^€^ | 334 | 10 | 67.68 | 1.73 | 10.22 | 8 | NA | 0.35 | 0.14 | 39 | 3.9 | 1.9 | 27.6 | 274 |
|  | *Gondli** | Little millet | *Panicum miliare* Lam. | 346 | 10.1 | 65.55 | 3.89 | 7.72 | 2 | NA | 0.26 | 0.05 | 36 | 1.2 | 1.8 | 16.1 | 130 |
|  | *Danbodi** | Cowpea, brown | *Vigna unguiculata* (L.) Walp. *^€^* | 365 | 24.2 | 61.9 | 2.3 | 28.6 | 10 | NA | 0.33 | 0.09 | 231 | 6.1 | 3.8 | 91 | NA |
|  | *Bodi** | Cow pea, white | *Vigna unguiculata* (L.) Walp. *^€^* | 320 | 21.3 | 53.77 | 1.14 | 11.7 | 8 | NA | 0.33 | 0.09 | 249 | 5.0 | 3.6 | 84.1 | 378 |
|  | *Rambada / Urad dal** | Black gram dal | *Vigna mungo* (L.) *^€^* | 324 | 23.1 | 51 | 1.69 | 11.93 | 10 | NA | 0.21 | 0.09 | 89 | 4.7 | 3 | 55.7 | 375 |
|  | *Sutri^$^* | Rice bean | *Vigna umbellata* (Thumb.) Ohwi & H. Ohashi*^€^* | 332 | 21.5 | 60.9 | 0.3 | NA | NA | NA | NA | NA | NA | NA | NA | 302 | 297 |
|  | *Kulthi** | Horse Gram, whole | *Macrotyloma uniflorum* (Lam.) Verdc. *^€^* | 321 | 22 | 57.2 | 0.5 | NA | 59 | NA | 0.32 | 0.24 | 163 | 8.8 | 2.7 | 269 | 298 |
|  | *Khesari dal^$^* | Khesari *Dal* | *Lathyrus sativus* L. | 345 | 28.2 | 56.6 | 0.6 | NA | NA | NA | NA | NA | NA | 6.3 | NA | 90 | 317 |
|  | *Baturi dal* ^¥^ | Munmuna | *Vicia hirsuta* (L.) Gray | 341 | 27 | 55.54 | ND | 8.79 | 178 | 12.45 | 0.22 | 1.15 | ND | 17.1 | 3.8 | 77.5 | 83.3 |
|  | *Saru ara* * | Colocasia leaves | *Colocasia esculenta* (L.) Schott*^€^* | 44 | 3.4 | 3.69 | 1.38 | 5.6 | 146 | 40.7 | 0.08 | 0.07 | 159 | 3.4 | 0.8 | 216 | 57.9 |
|  | *Kakaru ara** | Pumpkin leaves | *Cucurbita pepo* L. | 44 | 4.2 | 4.75 | 0.74 | 2.25 | 1455 | 12.3 | 0.07 | 0.13 | 34 | 5.58 | 0.9 | 217 | 64.5 |
|  | *Kantha ara^#^* | Kantha leaves | *Dentella repens* (L.) J.R. Forst. & G. Forst. | 46 | 3.5 | 8 | NA | 7.1 | 11680 | 9 | 3.07 | NA | 7 | 81.1 | 1.0 | 425 | NA |
|  | *Kaddu ara^$^* | Bottle gourd leaves | *Lagenaria siceraria* (Molina) Standl. *^€^* | 39 | 2.3 | 6.1 | 0.7 | NA | NA | NA | NA | NA | NA | NA | NA | 80 | 59 |
|  | *Muri/Munga ara** | Drumstick leaves | *Moringa oleifera* Lam. | 67 | 6.4 | 5.62 | 1.64 | 8.21 | 17542 | 108 | 0.06 | 0.45 | 43 | 4.6 | 0.7 | 314 | 109 |
|  | *Leped ara** | Amaranth leaves | *Amaranthus spinosus* L. | 24 | 1.6 | 2.01 | 0.45 | 2.21 | 1594 | 77.3 | 0.01 | 0.13 | 41 | 6.3 | 1.6 | 359 | 72.4 |
|  | *Garundi / Gundri ara ** | Ponnaganni | *Alternanthera sessilis* (L.) R.Br. ex DC. | 51 | 5.3 | 5.17 | 0.71 | 6.74 | 5288 | 103 | 0.02 | 0.1 | 48 | 3.9 | 1 | 388 | 53.2 |
|  | *Khadia ara^#^* | Dhurup leaves | *Leucas lavandulifolia* Sm. | 67 | 5.7 | 11.1 | NA | 6.7 | 18460 | 8 | NA | NA | 11 | 20.0 | 0.8 | 236 | NA |
|  | *Boot ara^$^* | Bengal gram leaves | *Cicer arietinum* L. | 97 | 7 | 14.1 | 1.4 | NA | NA | NA | NA | NA | NA | 23.8 | NA | 340 | 120 |
|  | *Hesa ara^#^* | Banyan leaves | *Ficus benghalensis* L. | 121 | 2.9 | 27.3 | NA | 22.3 | 8200 | NA | NA | NA | 4 | 2.8 | 0.8 | 295 | NA |
|  | *Lehsun ara^* | Garlic leaves | *Allium sativum* L*.* | 34 | 3.1 | 5.4 | NA | 4.9 | 5100 | 6 | NA | NA | 3 | 5.9 | 0.2 | 221 | NA |
|  | *Lupu ara^* | Chhaya / Kapurijari / Gorakhbuti | *Aerva lanata* (L.) Juss. Ex Schult. | 56 | 4.6 | 9.5 | ND | 5.9 | 21760 | 12 | ND | ND | 41 | 22.1 | 0.7 | 202 | ND |
|  | *Sing ara*^¥^ | Koinaar leaves | *Bauhinia purpurea* L. | 85 | 8.7 | 12.52 | ND | 4.22 | 2935 | 2.6 | 15.34 | 0.71 | ND | 4.3 | 1.3 | 147 | ND |
|  | *Epil ara/ /kudrum** | Gogu leaves, red stem | *Hibiscus sabdariffa* L. | 36 | 1.9 | 4.06 | 1.09 | 4.59 | 5285 | 29.7 | 0.13 | 0.06 | 75 | 7.7 | 0.7 | 145 | 42 |
|  | *Jojo ara** | Tamarind leaves | *Tamarindus indica* L. | 71 | 5.8 | 10.04 | 0.49 | 10.7 | 168 | 28.2 | 0.12 | 0.03 | 92 | 2.8 | 0.9 | 66.9 | 86.8 |
|  | *Chakod ara ^$^* | Pot Cassia | *Senna obtusifolia* (L.) H.S. Irwin & Barneby*^€^* | 49 | 5 | 5.5 | 0.8 | NA | 10512 | 82 | 0.08 | 0.19 | NA | 12.4 | NA | 520 | NA |
|  | *Kalmi ara^$^* | Water spinach | *Ipomoea aquatica* Forssk. *^€^* | 28 | 2.9 | 3.1 | 0.23 | NA | 1980 | 10 | 0.05 | 0.13 | NA | 3.9 | NA | 110 | NA |
|  | *Sarla ara^$^* | Katai leaves | *Meyna pubescens* (Kurz.) Robyns*^€^* | 86 | 4 | 14.9 | 1.1 | NA | NA | NA | NA | NA | NA | NA | NA | 127 | NA |
|  | *Beng saag/ Chokke ara^* | Beng leaves | *Centella asiatica* (L.) Urb*.* | 54 | 1.9 | 11 | ND | 7.5 | 500 | 5 | Nil | Nil | NA | 55.7 | 1.9 | 231 | NA |
|  | *Lal bhaji/Lal saag** | Amaranth, tender, red leaves | *Amaranthus retroflexus* L. *^€^* | 33 | 3.93 | 2.37 | 0.63 | 4.91 | 21449 | 86.2 | 0.01 | 0.26 | 82 | 7.2 | 1.3 | 245 | 75.9 |
|  | *Phutkal ara^* | Phutkal leaves | *Ficus virens* Aiton *^€^* | 324 | 2.4 | 58.4 | 1.8 | 3.5 | 30 | 4 | NA | NA | NA | 4.1 | 0.3 | 672 | NA |
|  | *Kakaru ara** | Pumpkin leaves | *Cucurbita maxima* L. | 44 | 4.2 | 4.75 | 0.74 | 2.25 | 1455 | 12.3 | 0.07 | 0.13 | 34 | 5.6 | 0.9 | 271 | 65.5 |
|  | *Susni ara/Chatom ara* ^¥^ | Sunsuni leaves | *Marsilea minuta* L*.* | 113 | 7.44 | 20.76 | ND | 7.24 | 15333 | 1.2 | 2.3 | 2.6 | ND | 16.6 | 3.5 | 90.1 | ND |
|  | *Ohio ara* ^¥^ | - | *Trianthema portulacastrum* L. | 26 | 2.5 | 3.97 | ND | 1.45 | 5200 | 1.2 | 22.47 | 16.63 | ND | 35.6 | 0.6 | 71.4 | ND |
|  | *Mattha ara* ^¥^ | Mata leaves | *Antidesma acidum* Retz. *^€^* | 109 | 4.79 | 22.38 | ND | 7.63 | 2871 | 1.2 | 13.87 | 2.62 | ND | 5.2 | 0.7 | 474.2 | ND |
|  | *Poi Saag* ^¥^ | Malabar spinach | *Basella alba* L. | 21 | 3.17 | 2.03 | ND | 1.63 | 62 | 2.9 | ND | ND | ND | 3.4 | 0.3 | 160.5 | 25.2 |
|  | *Aloo ara*^¥^ | Potato leaves | *Solanum tuberosum* L. | 38 | 6.29 | 3.22 | ND | 1.23 | 19851 | 3.6 | 0.5 | ND | ND | 7.2 | 0.3 | 127.9 | 0.02 |
|  | *Khesari*  *ara* ^¥^ | Khesari leaves | *Lathyrus sativus* L. | 46 | 6.23 | 5.2 | ND | 2.27 | 5452 | 1.2 | 1.9 | 0.46 | ND | 6.6 | 0.4 | 69.5 | 0.02 |
|  | *Dali/Dail ara*^¥^ | Nunia leaves | *Portulaca quadrifida* L. | 19 | 3.57 | 1.19 | ND | 4.85 | 1015 | 1.6 | 0.17 | 0.73 | ND | 31.2 | 0.8 | 10.8 | 0.2 |
|  | *Undku ara* ^¥^ | Kena leaves | *Commelina benghalensis* L. | 42 | 3.43 | 6.99 | ND | 4.25 | 1800 | 1.5 | 1.79 | 3.15 | ND | 21.9 | 0.7 | 121.2 | 0.2 |
|  | *Soredhe/ Bir/Rimil ara*^¥^ | Dheniani | *Olax scandens* Roxb. | 98 | 4.63 | 19.83 | ND | 2.87 | 1186 | 3.1 | 0.33 | 3.59 | 0.4 | 7.7 | 2.0 | 115.9 | 14.3 |
|  | *Budilaie ara*^¥^ | Khatta saag | *Cissus auriculata* Roxb.  *^€^* | 26 | 2.65 | 3.73 | ND | 2.42 | 92 | 1.7 | 6.24 | 0.16 | 0.2 | 1.9 | 0.8 | 257.7 | 10.3 |
|  | *Kauwa saag* ^¥^ | Kauwa leaves | *Rungia quinqueangularis* Koen. | 42 | 4.69 | 5.91 | ND | 1.97 | 287 | ND | ND | 0.66 | 0.4 | 8.2 | 18.7 | 340.4 | 21.7 |
|  | *Uri le ara*^¥^ | Purslane | *Portulaca oleracea* L. | 58 | 3.43 | 11.02 | ND | 5.62 | 249 | ND | 0.65 | 0.46 | 0.3 | 24.4 | 1.4 | 200.2 | 36.9 |
|  | *Sirgiti ara/Siliary ara* ^¥^ | Garkha/ Gadrya/ Garke | *Celosia argentea* L*.* | 48 | 4.58 | 6.41 | 0.4 | 5.93 | 1157 | ND | ND | 0.34 | 0.3 | 7.7 | 0.2 | 202.6 | 40.1 |
|  | *Kecho ara*^¥^ | Punarnava | *Boerhavia procumbens* Banks ex Roxb. *^€^* | 52 | 4.37 | 8.67 | ND | 2.47 | 2257 | 0.7 | 0.42 | 1.31 | 0.4 | 9.4 | 1.7 | 877.9 | 8.7 |
|  | *Charmani ara*^¥^ | Hurhura | *Cleome monophylla* L. | 42 | 4.71 | 5.89 | ND | 2.21 | 1128 | 3.2 | 0.14 | 0.57 | ND | 29.2 | 0.9 | 458.7 | 21.3 |
|  | *Piring ara*^¥^ | Netho saag | *Medicago denticulata* Willd..  *^€^* | 67 | 1.37 | 15.5 | ND | 7.39 | 3124 | ND | 1.04 | 0.65 | ND | 7.8 | 0.9 | 293.1 | 10.4 |
|  | *Tir ara/ Lochkor ara*^¥^ | Arrow head | *Sagittaria latifolia* L. | 51 | 2.95 | 12.43 | ND | 6.17 | 2561 | ND | 0.31 | 5.55 | 1 | 5.1 | 0.3 | 163.9 | 12.7 |
|  | *Sanga ara* ^¥^ | Sweet potato leaves | *Ipomoea batatas (*L.) Lam. | 51 | 2.64 | 10.26 | ND | 5.87 | 1717 | ND | 0.99 | 0.11 | ND | 0.3 | 0.1 | 3.5 | 9.2 |
|  | *Kotle ara* ^¥^ | Patsan | *Hibiscus cannabinus* L. | 42 | 1.54 | 9.07 | ND | 5.72 | 2665 | ND | 0.36 | 0.24 | ND | 2.1 | 0.1 | 177.9 | 10.9 |
|  | *Kohna/ Ketha ara* ^¥^ | Ash gourd leaves | *Benincasa hispida* (Thunb.) Cogn. | 34 | 3.46 | 5 | ND | 3.72 | 637 | ND | 0.23 | 0.83 | ND | 0.3 | 0.1 | 12.9 | 10.7 |
|  | *Chaari ara* ^¥^ | Kharika leaves | *Spergula pentandra* L. | 39 | 0.59 | 9.27 | ND | 6.97 | 83 | ND | 3.94 | 0.4 | 5 | 58.7 | 53.9 | 90.9 | ND |
|  | *Chiringid ara*^¥^ | Akra | *Vicia sativa* L . | 64 | 0.71 | 15.21 | ND | 7.75 | 2694 | ND | 1.8 | 6.77 | 8 | 6.7 | 0.7 | 52.5 | ND |
|  | *Bir chhatom ara*^¥^ | Khatti buti | *Oxalis corniculata* L. | 54 | 0.66 | 12.75 | ND | 7.94 | 2600 | ND | 1.61 | 0.93 | 6 | 2.8 | 2.3 | 192.4 | ND |
|  | *Bir Karela** | Bitter gourd | *Momordica dioica* Roxb. | 19 | 1.3 | 2.53 | 0.24 | 3.49 | 126 | 50.8 | 0.06 | 0.04 | 51 | 1.1 | 0.3 | 16.2 | 40.2 |
|  | *Manal/Simbi ^$^* | Field beans, tender | *Lablab purpureus* (L.) Sweet *^€^* | 48 | 3.8 | 7.2 | 0.1 | NA | 187 | 9 | 0.1 | 0.06 | NA | 0.8 | 0.4 | 210 | NA |
|  | *Jhinga/ Dodo/Doro ** | Ridge gourd, smooth skin | *Luffa acutangula* (L.) Roxb. | 15 | 1 | 2.24 | 0.13 | 1.85 | 349 | 8.1 | 0.02 | 0.01 | 27 | 0.5 | 0.2 | 14.9 | 39.3 |
|  | *Burju Baha* ^¥^ | Kachnar flower | *Bauhinia variegata* L. | 83 | 2.98 | 17.7 | ND | 8.49 | 416 | 2.4 | ND | 0.3 | ND | 3.4 | 0.7 | 404.9 | 447.7 |
|  | *Kundri** | Kovai | *Coccinia grandis* (L.) Voigt*^€^* | 19 | 1.2 | 2.41 | 0.24 | 3.25 | 147 | 21.1 | 0.04 | 0.02 | 50 | 0.2 | 0.1 | 37.1 | 26.3 |
|  | *Bodi ^#^* | Barbatti vegetable | *Vigna unguiculata* (L.) Walp. *^€^* | 49 | 3.7 | 8.5 | NA | 4.4 | 36 | 9 | NA | NA | 7 | 0.9 | 0.6 | 41 | NA |
|  | *Ketha^$^* | Ash gourd | *Benincasa hispida* (Thunb.) Cogn. | 10 | 0.4 | 1.9 | 0.1 | NA | NA | 1 | 0.06 | 0.01 | NA | 0.8 | NA | 30 | 20 |
|  | *Bans** | Bamboo tender | *Bambusa vulgaris* Schrad. ex J.C. Wendl. | 16 | 1.3 | 1.67 | 0.35 | 1.55 | NA | 15.7 | 0.06 | 0.06 | 17 | 0.3 | 0.3 | 10 | 39 |
|  | *Kutumba / Hanjen* ^¥^ | Kutma | *Solanum torvum*  Swartz. | 54 | 3.46 | 9.99 | ND | 5.42 | 24 | 1.5 | 1.28 | 2.01 | 1 | 0.6 | 0.2 | 29.8 | 16.4 |
|  | *Jiri ba^¥^* | Sanai phool | *Crotalaria juncea* L. | 120 | 2.9 | 27.04 | ND | 7.43 | 1112 | 1.77 | 3.09 | ND | ND | 7.6 | 0.2 | 320.2 | 537.2 |
|  | *Adel sanga** | Tapioca | *Manihot esculenta* Crantz. | 80 | 1.03 | 17.81 | 0.2 | 4.61 | NA | 17.6 | 0.07 | 0.02 | 26 | 0.8 | 0.1 | 25.9 | 42.6 |
|  | *Saaru** | Colocasia | *Colocasia esculenta* (L.) Schott. *^€^* | 90 | 3.3 | 17.85 | 0.17 | 3.22 | 7 | 1.8 | 0.06 | 0.03 | 20 | 0.7 | 0.4 | 30.1 | 81.1 |
|  | *Haranbho /Piski sanga* ^¥^ | Ban-aloo/ Gethia kanda | *Dioscorea bulbifera* L. | 44 | 2.13 | 8.97 | ND | 5.27 | ND | ND | 1.83 | 5.07 | 1 | 1.8 | 0.1 | 4.9 | 8.6 |
|  | *Haatikata/ Aaru/ Jat sanga* ^¥^ | Khamaloo/ Chupri-aloo | *Dioscorea alata* L. | 126 | 3.25 | 27.78 | 0.25 | 3.71 | 11 | 5.6 | 5.96 | 11.12 | ND | 3.8 | 0.4 | 10.7 | 28.1 |
|  | *Toti* ^¥^ | Pechki | *Colocasia esculenta* (L.) Schott. | 73 | 3.22 | 14.67 | 0.12 | 5.86 | ND | ND | 0.14 | 0.32 | ND | 1.4 | 0.2 | 1.8 | 10.9 |
|  | *Pindi* ^¥^ | Oal | *Amorphophallus paeoniifolius* (Dennst.) Nicolson | 64 | 6.32 | 9.65 | ND | 1.15 | ND | 3.1 | ND | ND | ND | 11.1 | 1.1 | 35.7 | 45.0 |
|  | *Haseaar sanga* ^¥^ | *-* | *Dioscorea quartiniana* A. Rich. *^€^* | 72 | 4.4 | 13.49 | ND | 1.34 | ND | 3.1 | 1.09 | ND | ND | 55.9 | 0.6 | 33.2 | 26.4 |
|  | *Amda^$^* | Ambada | *Spondias pinnata* (L.f.) Kurz. *^€^* | 48 | 0.7 | 4.5 | 3 | NA | NA | NA | NA | NA | NA | 3.9 | NA | 36 | 11 |
|  | *Mahua^$^* | Mahua, ripe | *Madhuca longifolia* (J. Koenig. ex L.) J.F. Macbr. | 111 | 1.4 | 22.7 | 1.6 | NA | 307 | 40 | NA | NA | NA | 0.2 | NA | 45 | 22 |
|  | *Soso ^$^* | Marking nut (kernel)/ Bhelwa | *Semecarpus anacardium* L.f. | 587 | 26.4 | 28.4 | 36.4 | NA | NA | NA | NA | NA | NA | 6.1 | NA | 295 | NA |
|  | *Godaari* * | Zizyphus | *Zizyphus jujuba* Mill. | 49 | 1.3 | 9.4 | 0.35 | 3.73 | 2 | 60.9 | 0.01 | 0.02 | 6 | 0.4 | 0.1 | 46.6 | 32.3 |
|  | *Tiril / Kendu ^$^* | Tumki | *Diospyros melanoxylon* Roxb. | 112 | 0.8 | 26.8 | 0.2 | NA | 361 | 1 | 0.01 | 0.04 | NA | 0.5 | NA | 60 | NA |
|  | *Tamras** | Taal Phal | *Borassus flabellifer* L. | 101 | 0.5 | 4.92 | 0.12 | 2.4 | NA | 0.2 | 0.01 | NA | 24 | NA | 0.1 | NA | NA |
|  | *Sinju ** | Wood apple | *Aegle marmelos*  (L.) Correa | 136 | 2.6 | 28.21 | 0.57 | 6.31 | 3 | 7.5 | 0.03 | 0.04 | 55 | 0.2 | 0.1 | 47.9 | 37.2 |
|  | *Baadi^$^* | Banyan fruit | *Ficus benghalensis* L. | 72 | 1.7 | 11.8 | 2 | NA | NA | NA | NA | NA | NA | NA | NA | 364 | NA |
|  | *Dahu* ^¥^ | Barhar | *Artocarpus lakoocha* Roxb. | 121 | 2.88 | 27.33 | ND | 7.2 | 1843 | 8.9 | 0.32 | 1.32 | ND | 1.8 | 0.1 | 54.7 | ND |
|  | *Kusum/Baru* ^¥^ | Kusum fruit | *Schleichera* oleosa (Lour.) Merr. | 144 | 6.31 | 29.72 | ND | 14.89 | 6238 | 3.1 | ND | ND | ND | 44.2 | 0.8 | 134.8 | 51.3 |
|  | *Loa/Dumur* ^¥^ | Gular | *Ficus racemosa* L.  *^€^* | 52 | 3.24 | 9.7 | ND | 5.82 | ND | ND | ND | ND | 1 | 1.5 | 0.4 | 84 | 10.3 |
|  | *Aanri* ^¥^ | Bhui-gular/ Khaina/ Khunia | *Ficus semicordata* Buch.-Ham. Ex Sm. | 57 | 3.17 | 10.76 | 0.13 | 6.25 | 9 | ND | ND | 0.18 | ND | 0.4 | 0.1 | 3.6 | 10 |
|  | *Rugda* ^¥^ | Mushroom | *Geastrum* | 138 | 4.86 | 29.53 | 0.06 | 7.37 | ND | ND | 0.58 | 0.37 | 0.2 | 6.8 | 3.1 | 193.4 | 30.2 |
|  | *Gitilud* ^¥^ | Mushroom | - | 67 | 3.52 | 13.17 | ND | 6.89 | 9 | ND | 0.19 | 0.17 | 3 | 10.8 | 0.6 | 9.6 | 24.3 |
|  | *Indiud* ^¥^ | Mushroom | *T. albuminosa* | 38 | 2.2 | 6.87 | 0.23 | 4.86 | ND | ND | 1.45 | 0.28 | 5 | 4.0 | 0.3 | 4.8 | 17.9 |
|  | *Koodeud/ Kundaud* ^¥^ | Mushroom | *Termitomyces clypeatus* | 41 | 2.49 | 7.45 | 0.19 | 6.13 | 9 | ND | 1.65 | 0.49 | 3 | 6.5 | 1.0 | 11.2 | 9.9 |
|  | *Loa suti ^$^* | Snail | *Pila globosa* | 97 | 10.5 | 12.4 | 0.6 | NA | NA | NA | NA | NA | NA | NA | NA | 870 | 116 |
|  | *Pothi hako/ Potha Hako^$^* | Puti fish | *Barbus* sp. | 106 | 18.1 | 3.1 | 2.4 | NA | NA | 15 | NA | NA | NA | 1 | NA | 110 | NA |
|  | *Redhayi / Mangri^$^* | Walking catfish | *Clarias batrachus* | 86 | 15 | 4.2 | 1 | NA | NA | NA | NA | NA | NA | 0.7 | NA | 210 | 290 |
|  | *Setua/*  *Keyosuti ^$^* | Mussel | *Margaritifera margaritifera* | 81 | 14.5 | 2.1 | 1.6 | NA | NA | NA | NA | NA | NA | NA | NA | 592 | NA |
|  | *Moosa/ Gudu^$^* | Field Rat | *Rattus argentiventer* | 104 | 23.6 | NA | 1 | 0.1 | NA | NA | NA | NA | NA | NA | NA | 30 | NA |
|  | *Jungli*  *murgi** | Wild Hen | *Galloanseres* sp. | 188 | 18.9 | NA | 12.9 | NA | 27 ^a^ | NA | 0.11 | 0.13 | 10 | 1.40 | 2.2 | 20 | 235 |
|  | *Kabutar** | Pigeon | *Columba livia domestica* | 126 | 17.9 | NA | 6.03 | NA | 15 ^a^ | NA | 0.18 | 0.39 | 8 | 3.8 | 2.4 | 18.1 | 255 |
|  | *Jangli suar ** | Wild Pig | *Sus scrofa* | 179 | 19.4 | NA | 11.3 | NA | 2 ^a^ | NA | 0.3 | 0.11 | 8 | 1 | 1.3 | 8.1 | 143 |
|  | *Demta/*  *Hau anda^$^* | Eggs of red ants | *Oceophylla smaragdina* | 131 | 13.4 | 9.1 | 4.6 | NA | NA | NA | NA | NA | NA | NA | NA | 104 | 107 |
|  | *Bale Machli^$^* | Bele fish | *Glossogoboius giuris* | 75 | 14.5 | 2.9 | 0.6 | NA | NA | 3 | NA | NA | NA | 1 | NA | 370 | 330 |
|  | *Ghaghar** | Common Quail | *Coturnix coturnix* | 138 | 20.9 | NA | 5.95 | NA | 13^a^ | NA | 0.05 | 0.24 | 9 | 1.90 | 1.13 | 20.6 | 300 |

***Note***: Text in Italics represents Mundari names

NA= Not available; ND= Not detected

^a^ Retinol expressed in µg/100gm for animal foods

^¥^ Laboratory analysis conducted as part of the study

**^β^** Scientific name cited from secondary literature: Singh and Kumar, 2016 (22); Singh and Kumar, 2014 (24); Singh and Kumar, 2015 (35); Singh LR, Rani V. 2019 (36); Longvah et al., 2017 (37 ); Gopalan et al., 1989 (38) ; Ghosh-Jerath et al., 2020 (39); Ghosh-Jerath et al.,2015 (42) and  *^€^* additionally verified from “Tropicos - Home,” 1982 (40) and “Home — The Plant List,” 2002 (41)

Secondary data on nutrient analysis: ^#^ Ghosh-Jerath et al., 2016 (19); *Longvah et al., 2017 (37); ^$^Gopalan et al., 1989 (38); ^ Ghosh-Jerath et al., 2015 (42)
